# Moderate expression and activity of flocculins underlie the characteristic flocculation phenotype of *Saccharomyces pastorianus*

**DOI:** 10.1128/aem.00883-26

**Published:** 2026-09-08

**Authors:** D. G. L. Ignacia, N. X. Bennis, C. Carqueija Cardoso, A. A. J. Potma, E. Knibbe, P. A. de Groot, I. Westerlaken, M. van den Broek, J.-M. G. Daran

**Affiliations:** 1 Department of Biotechnology, Delft University of Biotechnology 201231 , Delft, the Netherlands; University of Milano-Bicocca, Milan, Italy

**Keywords:** optogenetics, flocculation, gene expression, *Saccharomyces pastorianus*, lager brewing

## Abstract

Flocculation is a key technological trait in lager brewing, governing fermentation performance, yeast recovery, and beer quality. In the allo-aneuploid hybrid yeast *Saccharomyces pastorianus*, the genetic basis of flocculation remains poorly resolved due to its complex dual sub-genome architecture. Here, we systematically re-annotated and functionally characterized the complete *FLO* gene repertoire of the Group II strain CBS 1483. Thirteen *FLO* genes were identified, including allelic variants and a previously uncharacterized adhesin, Flo12, containing a Hyphal_reg_CWP domain instead of the canonical PA14 lectin-binding domain. Structural modeling revealed strong conservation of Ca²^+^-binding residues in PA14 domains, alongside repeat-region diversification likely contributing to functional variability. Using optogenetic expression in a *FLO*-null background, we demonstrated that *Spc*I-*FLO9*-1 and *Spc*I-*FLO9-2*_1 are the strongest drivers of flocculation, exhibiting NewFlo-like sugar sensitivity. Transcriptomic analysis during 17°P wort fermentation showed dynamic induction of these genes coinciding with flocculation onset. Surprisingly, deletion of both loci in CBS 1483 did not abolish but only delayed sedimentation in wort, accompanied by improved maltose utilization and attenuation. These findings reveal functional redundancy and compensatory mechanisms within the *FLO* network of lager yeast, highlighting the genetic complexity underlying flocculation, and providing a molecular framework to inform yeast selection, strain development, and optimization of the lager fermentation processes.

**IMPORTANCE:**

Flocculation, the process by which yeast cells aggregate and settle, is essential for producing clear, high-quality lager beer, and for efficient yeast recovery during brewing. However, the genetic basis of this trait in lager yeast has remained poorly understood because these strains possess unusually complex hybrid genomes. In this study, we systematically identified and characterized the complete set of flocculation genes in the industrial lager yeast *Saccharomyces pastorianus* CBS 1483. We demonstrated that lager yeast flocculation is not controlled by a single dominant gene, but instead emerges from the combined action of several moderately active adhesion proteins that are expressed at low levels during fermentation. Surprisingly, deleting the two strongest candidate genes only delayed, rather than eliminated, sedimentation, revealing a robust compensatory network that preserves brewing performance. These findings refine the current understanding of yeast flocculation and provide a molecular framework for developing brewing strains with improved fermentation efficiency, product consistency, and flavor quality.

## INTRODUCTION

Flocculation, the reversible, non-sexual and Ca^2+^-dependent aggregation of yeast cells into multicellular clusters (1), is a defining physiological trait of brewing yeasts and a crucial determinant of beer fermentation performance, product quality, and process efficiency (2). Brewing yeasts are traditionally classified as either top-fermenting ale strains, which associate with CO₂ bubbles and rise during fermentation, or bottom-fermenting lager strains, which aggregate and sediment at the end of fermentation (3). While flocculation is important across brewing traditions, it is especially critical in lager brewing, where the timely and consistent aggregation and sedimentation of bottom-fermenting yeasts is crucial to achieving predictable fermentation, efficient yeast recovery, and the characteristic clarity of lager beers (4).

The timely onset of flocculation is especially critical for efficient biomass removal. Premature yeast flocculation can lead to incomplete attenuation, particularly through insufficient consumption of maltotriose, a key late-stage sugar in wort, resulting in elevated residual sweetness and lower alcohol content (5, 6). Premature yeast flocculation may lead to elevated acetaldehyde levels and reduce late-formed alcohols and esters essential for a clean lager flavor profile (7). Early flocculation also limits yeast-mediated reduction of diacetyl to acetoin and 2,3-butanediol, leading to buttery off-flavors (8, 9). As lager yeasts often produce elevated sulfur compounds, particularly hydrogen sulfide, adequate yeast–beer contact time is required for their reabsorption or reduction (10–12). These processes depend on yeast metabolic activity, cell density, and fermentation duration (13).

Conversely, weak or delayed flocculation hampers clarification and yeast harvesting, prolongs conditioning through cold storage, and increases costs, downstream processing, and tank occupancy (14). Extended suspension also reduces yeast vitality for re-pitching and promotes autolysis-derived off-flavors. In large-scale breweries using cylindroconical fermenters, predictable flocculation yields significant economic benefits, as tank time is one of the major cost drivers in beer production (15). Therefore, moderate and predictable flocculation kinetics are essential for classic lager flavor profiles.

To ensure successful flocculation during brewing, it is critical to understand the factors that influence the onset and efficiency of flocculation. Flocculation phenotypes are shaped by a complex interplay of environmental, physiological, genetic, and epigenetic factors (16–34). At the molecular level, flocculation in yeast is primarily mediated by flocculins, cell wall–anchored proteins containing a lectin-like domain. In the presence of Ca²^+^, flocculins bind to mannose residues of α-mannan polysaccharides on glycosylated cell wall proteins of neighboring cells, thereby driving cell–cell adhesion and aggregate formation (35). These flocculins are encoded by the *FLO* adhesin gene family (36, 37). In *Saccharomyces cerevisiae*, *FLO* genes generally fall into two categories: *FLO1*, *FLO5*, *FLO9,* and *FLO10* are responsible for cell–cell adhesion, while *FLO11* is critical for pseudo-hyphal formation, invasive growth, substrate adhesion, and biofilm formation.

The expression of each *FLO* gene causes a unique phenotype, showing differences in floc morphology, sedimentation rate, sugar sensitivity, adhesion strength, and cell surface hydrophobicity (33, 38, 39). These can be broadly placed into three categories: The Flo1 phenotype, showing strong flocculation mainly inhibited by mannose; the NewFlo phenotype, which is inhibited by multiple sugars, including mannose, glucose, sucrose, maltose, and maltotriose that competitively bind to the carbohydrate-binding domain (33, 39–41); and the mannose-insensitive phenotype (42). Brewing strains often exhibit the preferable NewFlo phenotype (20, 40, 43), where flocculation can start only once the fermentable sugars have been nearly exhausted.

However, in the lager yeast *Saccharomyces pastorianus,* the situation is more complex. *S. pastorianus* is an allo-aneuploid interspecific hybrid yeast that originated from a natural hybridization between the domesticated ale yeast *S. cerevisiae* and the cold-tolerant wild species *Saccharomyces eubayanus* (44, 45). This hybridization has led to the evolution of two lineages, the Saaz (group I) and Frohberg (group II) strains, differing in the ploidy of each sub-genome complement and exhibiting variations in chromosome copy number across strains (46, 47). This hybrid nature has resulted in a high degree of genetic complexity, with large-scale genomic rearrangement and recombination between the two sub-genomes, leading to the development of novel genes and phenotypes not observed in the parental strains (28, 48, 49). Each sub-genome carries its own collection of *FLO* genes (46). With each strain showing its own unique genomic composition, understanding the role of *FLO* genes in *S. pastorianus* becomes an increasingly complicated endeavor.

To elucidate the molecular basis of flocculation in Group II lager yeast, we reanalyzed and compiled the complete *FLO* gene inventory of *S. pastorianus* CBS 1483 (46). After verifying gene integrity, the encoded protein sequences were analyzed for structure–function relationships. To support these *in silico* predictions, each identified flocculin was reintroduced individually into a *S. cerevisiae* platform strain lacking all native *FLO* genes (33) and placed under precise optogenetic control, enabling systematic dissection of their roles in floc formation, sugar sensitivity, and cell-surface hydrophobicity. Transcriptomic analysis of *S. pastorianus* CBS 1483 during wort fermentation then revealed which flocculins are expressed *in vivo*. Their contributions were validated through targeted gene deletions, and the resulting strains were phenotyped in standard wort fermentations.

Altogether, this molecular framework will inform yeast selection, strain development, and precision fermentation strategies, ensuring that lager production remains consistent, efficient, and aligned with the sensory expectations of brewers and consumers.

## MATERIALS AND METHODS

### Strains and growth conditions

All strains used in this study are listed in Table 1. The *Saccharomyces cerevisiae* strains used in this study originate from the CEN.PK background (50), while the S. *pastorianus* strains originate from the CBS 1483 background (46). Yeast stocks were stored in 30% vol/vol glycerol at −80°C. Yeast cultures were inoculated from frozen stocks into 500 mL shake flasks containing 100 mL liquid YPD medium containing 10 g·L^−1^ yeast extract, 20 g·L^−1^ peptone, and 20 g·L^−1^ glucose, and incubated at 30°C for *S. cerevisiae* and 20°C for *S. pastorianus* in an Innova 44 incubator shaker (Eppendorf, Nijmegen, the Netherlands) set at 200 rpm. For the flocculation tests, strains were inoculated in YPD. For the selection of transformants, YPD medium was supplemented with G418, nourseothricin, hygromycin B, or in combination at concentrations of 200 mg·L^−1^, 100 mg·L^−1^, and 100 mg·L^−1^, respectively. For selection of the pUDR868 (amdSYM marker), (NH_4_)_2_SO_4_ was replaced by 0.6 g·L^−1^ acetamide as a nitrogen source and 6.6 g L^−1^ K_2_ SO_4_ to compensate for sulfate in SMD, resulting in SMD-AC (51). The pH in all the media was adjusted to 6.0 with KOH. Solid media were prepared by adding 2% bacto agar to the abovementioned media. For the optogenetics experiments, an identical light setup was used for the induction of the *S. cerevisiae* strains containing an optogenetics circuit as previously described (33). Cultivations in E.B.C. tall tubes were performed in full malt wort (81 g·L^−1^ maltose, 24 g·L^−1^ maltotriose, 19 g·L^−1^ glucose, and 8 g·L^−1^ fructose) at 17°P (Heineken, Zoeterwoude, the Netherlands) at 12°C. The wort was supplemented with CaCl_2_ at a final concentration of 59.3 mg L^−1^.

**TABLE 1:** Strains used in this study

Strain name	Genotype	Parental strain	Origin
CEN.PK113-7D	MATα *MAL2-8C*		(50)
CBS 1483	Group II bottom-fermenting yeast, isolated from Heineken in July 1927		(44)
CDFM21L.1	*Saccharomyces eubayanus* isolated from Tibet		(52)
IMX2897	MATα *MAL2-8C* X 2::(p*TEF1*_VP16-EL222A79Q_t*CYC1*, pC120_QF2_t*ACT1*, p5xQUASf_*GFP*_t*ADH1*, p*CCW12*_QS_PSD_t*ENO1*) can1Δ::*Spycas9*-NatNT2 *flo1*Δ *flo5*Δ *flo9*Δ *flo10*Δ *flo11*Δ	IMK1060	(33)
IMX2912	MATα *MAL2-8C* X 2::(p*TEF1*_VP16-EL222A79Q_t*CYC1*, pC120_QF2_t*ACT1*, p5xQUASf_*Sc*I-*FLO1*_t*ADH1*, p*CCW12*_QS_PSD_t*ENO1*) can1Δ::*Spycas9*-NatNT2 *flo1*Δ *flo5*Δ *flo9*Δ *flo10*Δ *flo11*Δ	IMX2897	(33)
IMX3006	MATα *MAL2-8C* X 2::(p*TEF1*_VP16-EL222A79Q_t*CYC1*, pC120_QF2_t*ACT1*, p5xQUASf_*Spe*XII-*FLO1*-5_t*ADH1*, p*CCW12*_QS_PSD_t*ENO1*) can1Δ::*Spycas9*-NatNT2 *flo1*Δ *flo5*Δ *flo9*Δ *flo10*Δ *flo11*Δ	IMX2897	This study
IMX3007	MATα *MAL2-8C* X 2::(p*TEF1*_VP16-EL222A79Q_t*CYC1*, pC120_QF2_t*ACT1*, p5xQUASf_*Spe*VI*-FLO10*_t*ADH1*, p*CCW12*_QS_PSD_t*ENO1*) can1Δ::*Spycas9*-NatNT2 *flo1*Δ *flo5*Δ *flo9*Δ *flo10*Δ *flo11*Δ	IMX2897	This study
IMX3012	MATα *MAL2-8C* X 2::(p*TEF1*_VP16-EL222A79Q_t*CYC1*, pC120_QF2_t*ACT1*, p5xQUASf_*Spc*I*-FLO9*-1_t*ADH1*, p*CCW12*_QS_PSD_t*ENO1*) can1Δ::*Spycas9*-NatNT2 *flo1*Δ *flo5*Δ *flo9*Δ *flo10*Δ *flo11*Δ	IMX2897	This study
IMX3013	MATα *MAL2-8C* X 2::(p*TEF1*_VP16-EL222A79Q_t*CYC1*, pC120_QF2_t*ACT1*, p5xQUASf_*Spe*VI*-FLO12*_t*ADH1*, p*CCW12*_QS_PSD_t*ENO1*) can1Δ::*Spycas9*-NatNT2 *flo1*Δ *flo5*Δ *flo9*Δ *flo10*Δ *flo11*Δ	IMX2897	This study
IMX3101	MATα *MAL2-8C* X 2::(p*TEF1*_VP16-EL222A79Q_t*CYC1*, pC120_QF2_t*ACT1*, p5xQUASf_*Spc*I*-FLO9*-2_1_t*ADH1*, p*CCW12*_QS_PSD_t*ENO1*) can1Δ::*Spycas9*-NatNT2 *flo1*Δ *flo5*Δ *flo9*Δ *flo10*Δ *flo11*Δ	IMX2897	This study
IMX3102	MATα *MAL2-8C* X 2::(p*TEF1*_VP16-EL222A79Q_t*CYC1*, pC120_QF2_t*ACT1*, p5xQUASf_S*pc*I*-FLO9*-2_2_t*ADH1*, p*CCW12*_QS_PSD_t*ENO1*) can1Δ::*Spycas9*-NatNT2 *flo1*Δ *flo5*Δ *flo9*Δ *flo10*Δ *flo11*Δ	IMX2897	This study
IMX3104	MATα *MAL2-8C* X 2::(p*TEF1*_VP16-EL222A79Q_t*CYC1*, pC120_QF2_t*ACT1*, p5xQUASf_*Spe*I*-FLO1*-3_1_t*ADH1*, p*CCW12*_QS_PSD_t*ENO1*) can1Δ::*Spycas9*-NatNT2 *flo1*Δ *flo5*Δ *flo9*Δ *flo10*Δ *flo11*Δ	IMX2897	This study
IMX3105	MATα *MAL2-8C* X 2::(p*TEF1*_VP16-EL222A79Q_t*CYC1*, pC120_QF2_t*ACT1*, p5xQUASf_*Spe*I*-FLO1*-3_2_t*ADH1*, p*CCW12*_QS_PSD_t*ENO1*) can1Δ::*Spycas9*-NatNT2 *flo1*Δ *flo5*Δ *flo9*Δ *flo10*Δ *flo11*Δ	IMX2897	This study
IMX3109	CBS 1483 *Spc*I-*flo9*-2Δ::KanMX	CBS 1483	This study
IMX3185	CBS 1483 *Spc*I-*flo9*-1Δ::NatNT2 *Spc*I-*flo9*-2Δ::KanMX	CBS 1483	This study

### Molecular biological techniques

All primers designed in this study are shown in Table S1. The primers were ordered from Sigma-Aldrich (Burlington, MA, USA). RepliQa HiFi ToughMix (QuantaBio, Beverly, MA, USA) was used for obtaining fragments for sequencing and genomic integration according to the supplier’s instructions. For diagnostic purposes, PCR using DreamTaq PCR Master Mix (Thermo Fisher Scientific, Waltham, MA, USA) was utilized according to the supplier’s instructions. Plasmid isolations were performed using the GeneJET Miniprep kit (Thermo Fisher Scientific). All PCR products were purified from 1% TAE agarose using the Zymoclean Gel DNA Recovery Kit (Zymo Research, Irvine, CA, USA). PCR products were sequenced using Oxford Nanopore technology (Oxford Nanopore, Oxford, United Kingdom) by Plasmidsaurus (South San Francisco, CA, USA). All plasmids were propagated and stored using *E. coli* XL-1 Blue. Yeast genomic DNA for obtaining the *FLO* genes for integration and diagnostic purposes was isolated using the lithium acetate-SDS method (53).

### Plasmid construction

All plasmids used in this study are found in Table 2. For the targeting of *Spc*I-*FLO9*-1 and *Spc*I-*FLO9*-2, the gRNA expressing plasmids pUDP359 and pUDP361 were constructed using the pUDP002 plasmid and cloning strategy described by Juergens et al. (54). The plasmids pUD1506 and pUD1584 contain the single-guide RNA (sgRNA) sequences targeting *Spc*I-*FLO9*-1 and both alleles of *Spc*I-*FLO9*-2, respectively, flanked by a hammerhead ribozyme at the 5′ end, a hepatitis delta virus ribozyme at the 3′ end, and inward-facing BsaI sites on both sides for integration into pUDP002. The insertion of the gRNA sequences into the pUDP002 backbone was done using Golden Gate reactions as described by Juergens et al. (54). Insertion into the pUDP002 backbone was diagnosed using primers 11200/11201. All plasmids were sequenced using Oxford Nanopore Technology (Oxford Nanopore) by Plasmidsaurus.

**TABLE 2:** Plasmids used in this study

Name	Relevant characteristics	Purpose	Reference
pUDR868	2μm *ori bla amdSYM* gRNA*-GFP* gRNA*-GFP*	Expression of gRNA targeting *GFP*	(33)
pUDP002	*ori ampR* panARS^OPT^*AgTEF1*p-*hph-AgTEF1*t *ScTDH3*p-^BsaI-BsaI^-S*cCYC1*t *AaTEF1*p-*Spcas9*^D147Y P411T^-*ScPHO5*t	Expression of Cas9 and gRNA	(54)
pUD1506	*ori KanR*^BsaI^HH-*Sp*cI-*FLO9*-1-grna1-HDV^BsaI^	Contains gRNA targeting *Spc*I-*FLO9*-1	GeneArt
pUD1584	*ori KanR*^BsaI^HH-*Sp*cI-*FLO9*-2-grna1-HDV^BsaI^	Contains gRNA targeting *Spc*I-*FLO9*-2	GeneArt
pUDP359	*ori ampR* panARS^OPT^*AgTEF1*p-hph-*AgTEF1*t *ScTDH3*p-HH-*Spc*I-*FLO9*-1-grna1-HDV-*ScCYC1*t *AaTEF1*p-Spcas9^D147Y P411T^-*ScPHO5*t	Expression of Cas9 and gRNA targeting *Spc*I-*FLO9*-1	This study
pUDP361	*ori ampR* panARS^OPT^*AgTEF1*p-hph-*AgTEF1*t *ScTDH3*p-HH-*Spc*I-*FLO9*-2-grna1-HDV-*ScCYC1*t *AaTEF1*p-Spcas9^D147Y P411T^-*ScPHO5*t	Expression of Cas9 and gRNA targeting *Spc*I-*FLO9*-2	This study
pMEL13	2 μm *bla* KanMX gRNA-*CAN1*.Y	Source of KanMX cassette	(55)
pMEL15	2 μm *bla* NatNT2 gRNA-*CAN1*.Y	Source of NatNT cassette	(55)

### Strain construction

#### Construction of Opto-*FLO* strains

The construction of the CEN.K113-7D strains expressing *FLO* genes under optogenetic control was done according to the previously described workflow (33).

Each *FLO* gene was PCR amplified as a repair fragment from the genomic DNA of CBS 1483 using primers that introduced 60 bp flanks homologous to the 5xQUASfp and *ADH1t* regions of the OptoQ-AMP5 circuit to allow for integration using Cas9-mediated homologous recombination. IMX2897 was transformed with 500 ng of repair fragment and pUDR868 containing a gRNA targeting the GFP locus in the OptoQ-AMP5 system. This resulted in the strains IMX3006 (*Spe*XII-*FLO1*-5), IMX3007 (*Spe*VI*-FLO10*), IMX3012 (*Spc*I*-FLO9*-1), IMX3013 (*Spe*VI*-FLO12*), IMX3101 (*Spc*I*-FLO9*-2_1), IMX3102 (S*pc*I*-FLO9*-2_2), IMX3104 (*Spe*I*-FLO1*-3_1), and IMX3105 (*Spe*I*-FLO1*-3_2).

A nested PCR was performed to obtain the open reading frames for *Spc*I-*FLO9*-2, *Spe*I-*FLO1*-2, and *Spe*I-*FLO1*-3 using primer combinations 12145/12146, 20961/19618, and 12150/19618, respectively.

The primer pairs 20407/20708, 20918/21654, 20411/20412, 20413/20414, 20745/20746, 20747/20748, 21653/21654, and 21652/21679 were used to amplify the repair fragments for *Spc*VI-*FLO1*-1, *Spe*I-*FLO1*-3*, Spe*VI-*FLO12, Spc*I-*FLO9*-1*, Spc*VI-*FLO10, Spc*XII-*FLO1*-5*, Spe*XI-*FLO1*-4, and *Spc*I-*FLO9*-2, respectively, from genomic DNA or nested PCR amplicons. Successful integration into the optoQ-AMP5 circuit was confirmed via PCR using the primer pair 14455/19907 and sequencing by Plasmidsaurus. The amplicons for *Spc*VI-*FLO1*-1, *Spe*I-*FLO1*-2, and *Spe*XI*-FLO1*-4 were only used for sequencing.

#### Knockout of SpcI-FLO9-1 and SpcI-*FLO9-2* in CBS 1483

To knock out *Spc*I*-FLO9*-1 and both alleles of *Spc*I-*FLO9*-2 in CBS 1483, antibiotic resistance cassettes were obtained via PCR with 60-bp flanking regions homologous to the promoter and terminator of the *FLO* genes. For *Spc*I-*FLO9*-1, the primer pairs 21638/21639 and 22083/22084 were used to amplify the KanMX and NatNT2 markers from pMEL13 and pMEL15 (55), respectively, while for *Spc*I-*FLO9*-2, the primer pairs 21646/21647 were used for the amplification of the KanMX marker from pMEL13. CBS 1483 was transformed with 500 ng of pUDP361 and 1 µg of the KanMX resistance cassette to create the single knockout strain IMX3109 (*Spc*I-*flo9*-1Δ::KanMX) via electroporation as previously described (56). Then, the strain IMX3109 was transformed with 500 ng of pUDP359 and 1 µg of the NatNT2 cassette to construct the strain IMX3185 (*Spc*I-*flo9*-1Δ::KanMX, *Spc*I-*flo9*-2Δ::NatNT2). Successful integration of the KanMX cassettes into the *Spc*I*-FLO9*-1 locus was confirmed using the primer pairs 21615/3811, while the correct integration of the NatNT2 cassette into the *Spc*I-*FLO9*-1 locus was confirmed using the primer pairs 21615/21616.

### Fermentation in tall E.B.C. tubes

Fermentations in tall European Brewing Convention tubes (E.B.C.) (57) were performed using *S. pastorianus* strains CBS 1483 and IMX3185 as previously described by Bennis et al. (58). The fermentations were run in duplicates and were stopped after 16 days. Cell count was measured using a Nucleocounter YC-100 (Chemometec A/S, Allerød, Denmark), and wort density was measured using a DMA 35 basic handheld density meter (Anton Paar, Graz, Austria).

### RNA extraction and transcriptomics

The tall E.B.C. tube fermentation of CBS 1483 was sampled at days 1, 2, 5, 6, 9, 12, and 15 for RNA extraction. The total RNA was extracted, and the purity, integrity, and concentrations of the samples were determined as previously described (33, 59). cDNA libraries were prepared using the TruSeq RNA v2 kit (Illumina, San Diego, CA, USA). Paired-end sequencing of twofold 150 bp was performed on a 300 bp PCR-free insert library using a NovaSeq system (Illumina) at Macrogen (Amsterdam, the Netherlands). Duplicate biological samples were prepared and sequenced, generating an average of 20.5 million reads per sample. The sequencing reads were processed as previously described (60). The CBS 1483 assembly (46) was used as a reference. To differentiate between the allelic variants of *Spc*I-*FLO9*-2, allele-specific SNP variation positioned at 21 bp upstream of the 3′ end of the gene was quantified by viewing the bam files in IGV, and the ratio of each allele was calculated as a percentage over the total amount of reads at that location for all samples. This percentage was then used to calculate the fraction of reads belonging to each allele over the total amount of mapped reads at the target locus. Normalization was performed using the trimmed mean of M (TMM) method (61) for between-sample comparisons and reads per kilobase of transcript per million reads mapped (rpkm) for in-sample comparisons (62, 63). Differential expression was calculated from TMM values using the EdgeR package (63). The *Spc*XIV-*ALG9* gene, which was shown to have stable expression across a wide range of conditions, was used as a reference (64). Transcriptome RNA seq data of CBS 1483 grown in 17^o^P wort are available at NCBI under BioProject PRJNA1441968.

### Flo sequence analysis

The DNA and protein sequences of the *FLO* genes of *Saccharomyces cerevisiae* CEN.PK113-7D (BioProject [http://www.ncbi.nlm.nih.gov/bioproject/] accession PRJNA393501 [65]), *Saccharomyces eubayanus* CDFM21L.1 (accession PRJNA528469 [60]), and *Saccharomyces pastorianus* CBS 1483 (accession PRJNA266750 [46]) were obtained from their respective annotated assemblies. The protein sequences were analyzed according to the previously described workflow (33). The following nomenclature was used to discern between the multiple *FLO* genes across the three *Saccharomyces* species and sub-genomes: *S*(*p*) *c* I ‒ *FLO9 ‒ 2*_1. The first letter, *S*, indicates the *Saccharomyces* genus. *Saccharomyces pastorianus* is indicated with a *p*. The species or subgenome is indicated with either a *c* for *cerevisiae* or an *e* for *eubayanus*, followed by the chromosome on which the locus is located (“I” in this example). Finally, the gene name is indicated (*FLO9* in this example), followed by the copy number found in the genome (2 in this example) and allele (1 in this example), if applicable.

### Protein structural modeling

Full-length Flo proteins with the signal peptide trimmed were modeled using AlphaFold 3 (66). Structures were simulated in the presence of Ca²^+^ and mannose-α1,2-mannose-α1,2-mannose. Structural models were visualized using the PyMOL Molecular Graphics System, Version 3.0.3 (Schrödinger, LLC, New York, NY, USA).

### Microscopy

Image acquisition was conducted using a Zeiss AXIO Imager.Z1 microscope (Carl Zeiss AG, Oberkochen, Germany). The floc structure of the induced strains IMX3012 (*Spc*I*-FLO9-*1), IMX3013 (*Spe*VI-*FLO12*), IMX3101 (*Spc*I*-FLO9-*2_1), and IMX3102 (*Spc*I*-FLO9*-2_2) was examined by taking samples from the sedimented cells following the flocculation assay. Microscopic analysis of fractions was performed using Zeiss ZENBlue software (Carl Zeiss AG). Cells were imaged at 200× and 400× magnification for floc size analysis. The area of the flocs was measured using ImageJ as a measure of particle size. All particles smaller than 90 μm^2^ were filtered out to remove aggregates smaller than two aggregated budding cells. Statistical significance was calculated by comparing the geometric means using a lognormal one-way ANOVA test. The number of cell aggregates analyzed per strain ranged from 59 to 147. The data for CEN.PK113-7D, IMX2897 (*floΔ*), and IMX2912 (*Sc*I-*FLO1*) were obtained from our previous study (33).

### Flocculation assays and hydrophobicity assessment

For the flocculation assays and hydrophobicity assessments, the same methods as previously described (33) were utilized. The flocculation coefficient was determined by calculating the difference in ratio between the optical density (OD_660_) of deflocculated cells (A) and samples that were supplemented with CaCl_2_ to a concentration of 1% (B).


$$\frac{A-B}{A}\times 100=\text{Flocculation coefficient}$$

For the sugar inhibition assays, the flocculation buffer was supplemented with 1 M of sugars, with the exception of maltotriose at 500 mM. In the case of the cell surface hydrophobicity assessment, the difference in ratio between the OD_660_ of deflocculated cells incubated with (C) and without (A) the addition of dodecane was used. The percentage of hydrophobicity was calculated as the relative change in absorbance of the aqueous layer before and after vortexing. The OD_660_ was determined using a Jenway 7200 scanning spectrophotometer (Cole-Parmer Inc, Chicago, IL, USA).


$$\frac{A-C}{A}\times 100=\text{Hydrophobicity percentage}$$

### Whole-genome sequencing

The genomic DNA of the strain IMX3185 was isolated using the QIAGEN Genomic DNA isolation buffer set in combination with the Genomic-tip 100/G columns (Qiagen, Hilden, Germany) according to the manufacturer’s instructions. The concentration, integrity, and purity of the samples were determined using the Qubit 2.0 (Thermo Fisher Scientific), TapeStation 2200 (Agilent Technologies), and Nanodrop (Thermo Fisher Scientific), respectively. All genomic DNA samples were sequenced using Nanopore technology (Oxford Nanopore, Oxford, United Kingdom). Sequencing data are available at the NCBI repository (https://www.ncbi.nlm.nih.gov/) under BioProject PRJNA1441332 (Table S2). The raw Illumina reads were mapped to the CBS 1483 (46) genome using the Burrows-Wheeler (BWA) tool (23) and further processed using SAMtools (67). For mapping of IMX3185 reads, an extra contig was added to match the expected genomic integration of the antibiotic resistance cassettes for each strain. Chromosomal copy number was estimated by the Magnolya algorithm (68). The sequences were analyzed by visualizing the bam files in the Integrative Genomics Viewer (IGV) software (version 2.8.9) (69).

### Analytical measurements

Fermentable sugars (glucose, fructose, sucrose, maltose, and maltotriose) and ethanol concentrations were measured by HPLC (Agilent Technologies, 1260 HPLC system) equipped with a Bio-Rad HPX-42A column (300 × 7.8 mm, 25 μ) (Bio-Rad Laboratories, Hercules, CA, USA) and a 1260 Refractive Index Detector. The column was operated at 75 °C, and the elution was performed using milliQ at 0.5 mL·min^−1^ for 30 min. Compounds were measured using a RID at 35°C. Samples were 0.2 μm filter-sterilized before analysis (58, 70).

## RESULTS

### Composition of the *FLO* gene complement in *S. pastorianus* CBS 1483

Genomic re-analysis of *Saccharomyces pastorianus* CBS 1483 revealed the presence of 11 putative *FLO* genes (*Spc*I-*FLO9*-1, *Spc*I-*FLO9*-2, *Spc*VI-*FLO1*-1, *Spc*IX-*FLO11*-1, *Spe*I-*FLO1*-2, *Spe*I-*FLO1*-3, *Spe*VI-*FLO10*, *Spe*VI-*FLO12*, *Spe*IX-*FLO11*-2, *Spe*XI-*FLO1*-4, and *Spe*XII-*FLO1*-5, see Materials and Methods for gene nomenclature) (46) (Fig. 1). These genes were named based on the sequence similarity of their encoded proteins to *S. cerevisiae* Flo proteins and the presence of characteristic flocculin motifs (Table 3). In the CBS 1483 assembly (ASM1102231v1) (46), several mutations were identified in *FLO* genes that introduced frameshifts and premature stop codons. However, systematic amplicon resequencing demonstrated that many of these apparent mutations originated from sequencing errors in the published assembly. Resequencing led to the correction of five genes: *Spc*I-*FLO9*-1, *Spc*I-*FLO9*-2, *Spe*I-*FLO1*-2, *Spe*I-*FLO1*-3, and *Spe*VI-*FLO12,* which, with the exception of *Spe*I*-FLO1*-2, were found to encode full-length proteins. In addition, two allelic variants of *Spc*I-*FLO9*-2 and *Spe*I-*FLO1*-3 were identified and designated *Spc*I-*FLO9*-2_1 and _2*,* and *Spe*I-*FLO1-*3_1 and _2. The *Spc*I-*FLO9*-2 alleles differed in length by 522 bp, accounted for by a large difference in repeat type 1 and repeat type 2 counts, whereas the *Spe*I*-FLO1*-3 alleles were identical in length but exhibited 49 single-nucleotide variations, resulting in 17 amino acid substitutions (Table 3; Fig. S1). In total, 13 unique *FLO* genes were predicted, of which 4 are assumed to be non-functional due to premature stop codons: *Spc*VI-*FLO1*-1, *Spc*IX-*FLO11-*1, *Spe*I-*FLO1*-2, and *Spe*XI-*FLO1-*4 (Table 3). For comparison, the *FLO* gene complement of *S. eubayanus* CDFM21L.1 (52, 60) was also examined. Seven *FLO* genes were identified (Table 3): *Se*I*-FLO1*-1, *Se*I-*FLO1*-2, *Se*X-*FLO11*, *Se*VI-*FLO12*, *Se*VI*-FLO10*, *Se*XI-*FLO1*-3, and *Se*XII-*FLO1*-4. Among these, only *Se*XI-*FLO1*-3 contained a premature stop codon, resulting in a truncated protein.

**Fig 1:**
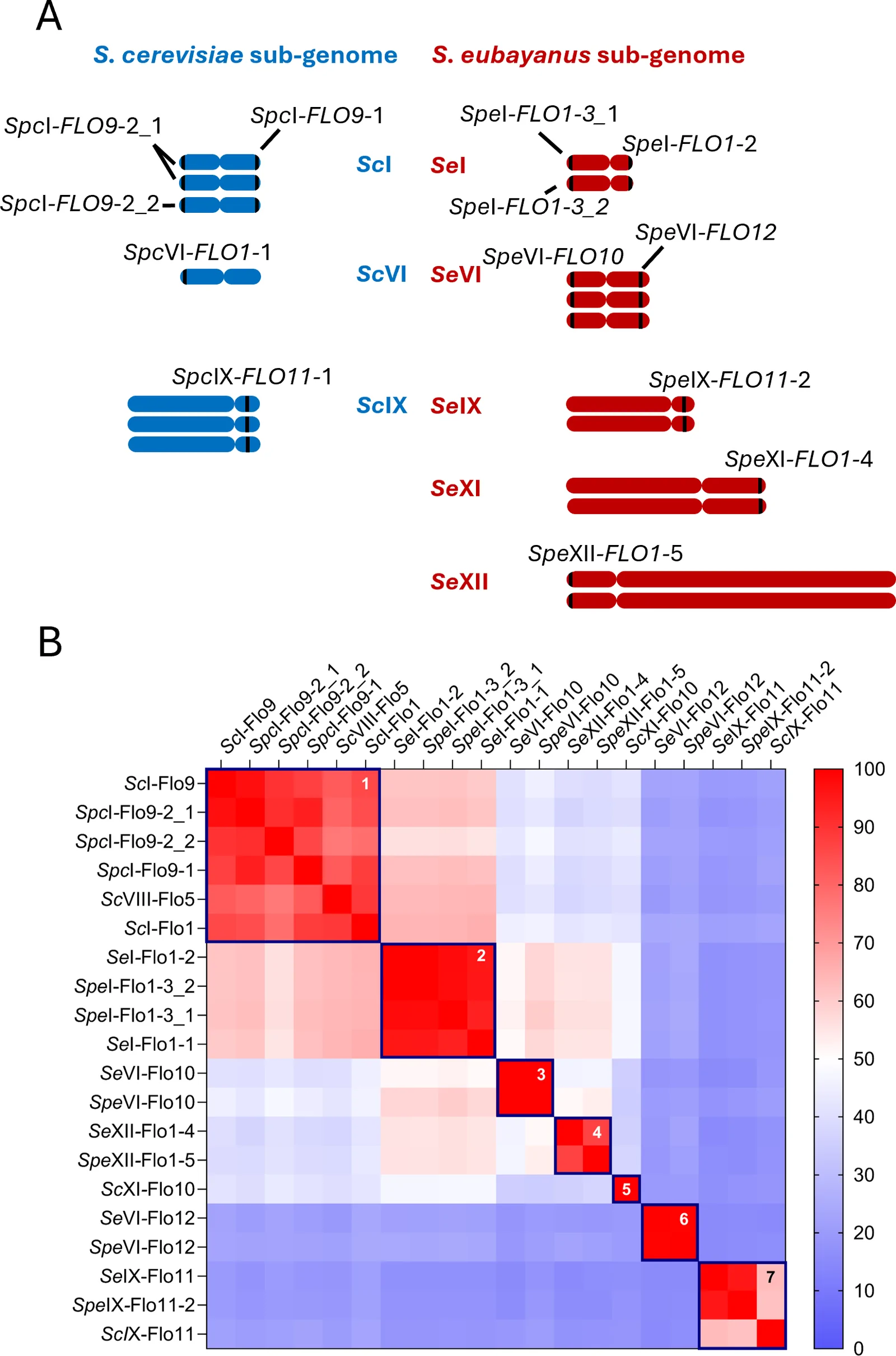
(**A**) Simplified depiction of chromosomal locations of all *FLO* genes identified in the CBS 1483 genome. With the exception of the *FLO11* genes, all *FLO* genes are located in the telomeric region. *FLO* genes are indicated with a black line on the chromosomes. (**B**) Multiple sequence alignment of full-length Flo proteins encoded in *S. cerevisiae* CEN.PK113-7D, *S. pastorianus* CBS 1483, and *S. eubayanus* CDFM21L.1 using Clustal Omega (71).

**TABLE 3:** Overview of relevant protein features of Flo proteins identified in *S. pastorianus* CBS 1483, *S. cerevisiae* CEN.PK113-7D (33), and *S. eubayanus* CDFM21L.1^*a*^

Species	Strain	Protein	Full ORF	Length (AA)	Signal sequence	Ligand-binding domain	Type	Central domain	GPI anchor site (homology)	GPI anchor site (NetGPI)	Repeat type 1	Repeat type 2	Repeat type 3	Repeat type 4	Repeat type 5	Repeat type 6	Repeat type 7
*S. pastorianus*	CBS 1483	*Spc*I*-*Flo9-1	Yes	988	1–24	113–248	PA14	274–498	G964	N967	5	2	6	2	16	-	-
*S. pastorianus*	CBS 1483	*Spc*I*-*Flo9-2_1	Yes	1,043	1–24	112–248	PA14	274–543	G1019	N1022	6	4	5	2	14	-	-
*S. pastorianus*	CBS 1483	*Spc*I*-*Flo9-2_2	Yes	869	1–24	112–248	PA14	274–318	G845	N848	1	7	6	2	12	-	-
*S. pastorianus*	CBS 1483	*Spc*VI-Flo1-1	No	345	1–23	119–255	PA14	-	-	-	1	-	-	-	-	-	-
*S. pastorianus*	CBS 1483	*Spc*IX-Flo11-1	No	350	1–21	43–193	Flo11	-	-	-	4*	-	-	-	-	-	-
*S. pastorianus*	CBS 1483	*Spe*I-Flo1-2	No	338	1–22	112–248	PA14	-	-	-	1	-	-	-	-	-	-
*S. pastorianus*	CBS 1483	*Spe*I-Flo1-3_1	Yes	753	1–22	111–247	PA14	274–421	G730	S727	2	-	3	1	16	-	-
*S. pastorianus*	CBS 1483	*Spe*I-Flo1-3_2	Yes	753	1–22	111–247	PA14	274–421	G730	S727	2	-	3	1	16	-	-
*S. pastorianus*	CBS 1483	*Spe*VI-Flo10	Yes	1,125	1–20	108–249	PA14	381–624	G1102	N1105	18*	-	3	-	-	-	-
*S. pastorianus*	CBS 1483	*Spe*VI-Flo12	Yes	1,253	1–17	9–298	HRCWD	417–752	G1231	S1227	1	-	1	-	-	21*	4*
*S. pastorianus*	CBS 1483	*Spe*IX-Flo11-2	Yes	1,302	1–21	57–207	Flo11	293–946	G1281	G1281	45*	5*	-	-	-	-	-
*S. pastorianus*	CBS 1483	*Spe*XI-Flo1-4	No	352	1–22	129–262	PA14	-	-	-	1	-	-	-	-	-	-
*S. pastorianus*	CBS 1483	*Spe*XII-Flo1-5	Yes	963	1–23	103–251	PA14	285–329	G940	G940	1	-	2	1	22	5	-
*S. cerevisiae*	CEN.PK113-7D	*Sc*I-Flo1	Yes	1,675	1–24	112–248	PA14	274–1173	G1652	G1652	20	2	3	5	13	-	-
*S. cerevisiae*	CEN.PK113-7D	*Sc*I-Flo9	Yes	959	1–24	112–248	PA14	274–453	G936	G936	4	4	3	2	13	-	-
*S. cerevisiae*	CEN.PK113-7D	*Sc*VIII-Flo5	Yes	1,075	1–24	112–247	PA14	274–633	G1052	G1052	8	2	3	2	6	-	-
*S. cerevisiae*	CEN.PK113-7D	*Sc*IX-Flo11	Yes	1,367	1–24	31–207	Flo11	313–879	G1346	G1346	41*	7*	2*	-	-	-	-
*S. cerevisiae*	CEN.PK113-7D	*Sc*XI-Flo10	Yes	1,169	1–24	123–270	PA14	302–606	G1146	N1154	10*	2*	3	2*	-	-	-
*S. eubayanus*	CDFM21L.1	*Se*I-Flo1-1	Yes	753	1–24	111–247	PA14	274–422	G730	S727	2	-	3	-	15	-	
*S. eubayanus*	CDFM21L.1	*Se*I-Flo1-2	Yes	753	1–22	111–247	PA14	274–422	G730	S727	2	-	3	-	15	-	-
*S. eubayanus*	CDFM21L.1	*Se*IX-Flo11	Yes	1,203	1–21	43–193	Flo11	293–847	G1182	G1182	35*	5*	-	-	-	-	-
*S. eubayanus*	CDFM21L.1	*Se*VI-Flo12	Yes	1,045	1–17	9–298	HRCWD	417–544	G1022	S1019	1	-	1	-	-	8*	4*
*S. eubayanus*	CDFM21L.1	*Se*VI-Flo10	Yes	997	1–20	99–262	PA14	381–547	G974	N977	11*	-	2	-	-	-	-
*S. eubayanus*	CDFM21L.1	*Se*XI-Flo1-3	No	36	1–22	99–263	PA14	-	-	-	-	-	-	-	-	-	-
*S. eubayanus*	CDFM21L.1	*Se*XII-Flo1-4	Yes	1,021	1–23	98–259	PA14	285–329	G998	G998	1	-	2	1	35	5	-

^*a*^ Relevant domains were identified using the InterPro database (72). Repeating motifs were identified using RADAR, Clustal Omega 1.2.4, and manual curation as previously described (33). The central domain consists of the repeat type 1 repeating motifs. The GPI anchor site was determined based on sequence alignment and homology with the known sites in *S. cerevisiae* flocculins. GPI-anchor site residues were also predicted using NetGPI (73), showing a discrepancy between tool- and homology-based predictions. Repeating motifs that deviate from the Flo1 type repeats are marked with an asterisk (*). Notably, Flo12 contains an alternative ligand-binding domain, the Hyphally Regulated Cell Wall Domain (HRCWD), two unique repeating motifs, and shares the Flo1 repeat type 1 and type 3 motifs. *Spe*XII-Flo1-5 and *Se*XII-Flo1-4 contain a unique repeat type 2. The *S. eubayanus* Flo1-like flocculins generally lack the repeat types 2 and 4.

Protein sequence alignment showed that the predicted flocculins could be broadly classified into four groups: Flo1-like (including Flo5 and Flo9, boxes 1 and 2), Flo10-like (boxes 3, 4, and 5), a novel Flo12-like group (box 6), and the Flo11-like (box 7) (Fig. 1B). Box 1 (Fig. 1; Table S3) contains *S. cerevisiae* Flo1-like flocculins, while box 2 contains the *S. eubayanus* Flo1-like proteins. Boxes 3 and 4 comprise proteins that share partial similarity with the Flo1-like flocculins but are more divergent; this group includes *S. cerevisiae* Flo10 and Flo proteins encoded by genes located on chromosomes VI and XII of *S. eubayanus,* along with their corresponding homologs in CBS 1483. Notably, box 6 includes a novel, distinct hypothetical flocculin designated Flo12, which exhibits structural similarity but limited identity compared to other flocculins. Finally, box 7 includes the Flo11-like proteins.

### Structure of *S. pastorianus* flocculins

Flo proteins are well known to contain three functional domains: an N-terminal carbohydrate-binding PA14 domain, a central serine/threonine-rich tandem repeat region that stabilizes the PA14 domain and stems it toward the extracellular space, and a C-terminal GPI anchor required for anchoring the protein to the cell wall (74–78). The domain structure of the 13 identified flocculins was analyzed (Table 3; Fig. S2).

### PA14 site alignment

The N-terminal carbohydrate-binding domain of flocculins belongs to the PA14 domain family (Pfam: PF07691) (79) and contains conserved Ca^2+^ coordinating sub-domains required for mannoside binding (75, 77, 80–82). Flo11 proteins share a similar overall architecture but contain a distinct “Flo11” domain (Pfam: PF10182) (38, 83) and were therefore excluded from our analyses.

Multiple sequence alignment of flocculins from *S. pastorianus* CBS 1483, *S. eubayanus* CDFM21L.1, and *S. cerevisiae* CEN.PK113-7D showed strong conservation of residues essential for Ca^2+^ and carbohydrate recognition (Fig. 2). The Flo1 subdomain was conserved across Flo1-like proteins, except in *Sc*IX-Flo10 and the flocculins encoded on CHRVI and CHRXII in CBS 1483 and CDFM21L.1. Key residues within the Flo1 subdomain (Q98) and the L3 loop (K194–I204) were maintained (84). Ca^2+^-binding loop 1 (CBL1; residues 157–161), including the D*cis*D motif (D160–D161), which is critical for Ca^2+^ docking (74), was conserved in all proteins except *Se*XII-Flo1-4, which contains a conservative D to E substitution.

**Fig 2:**
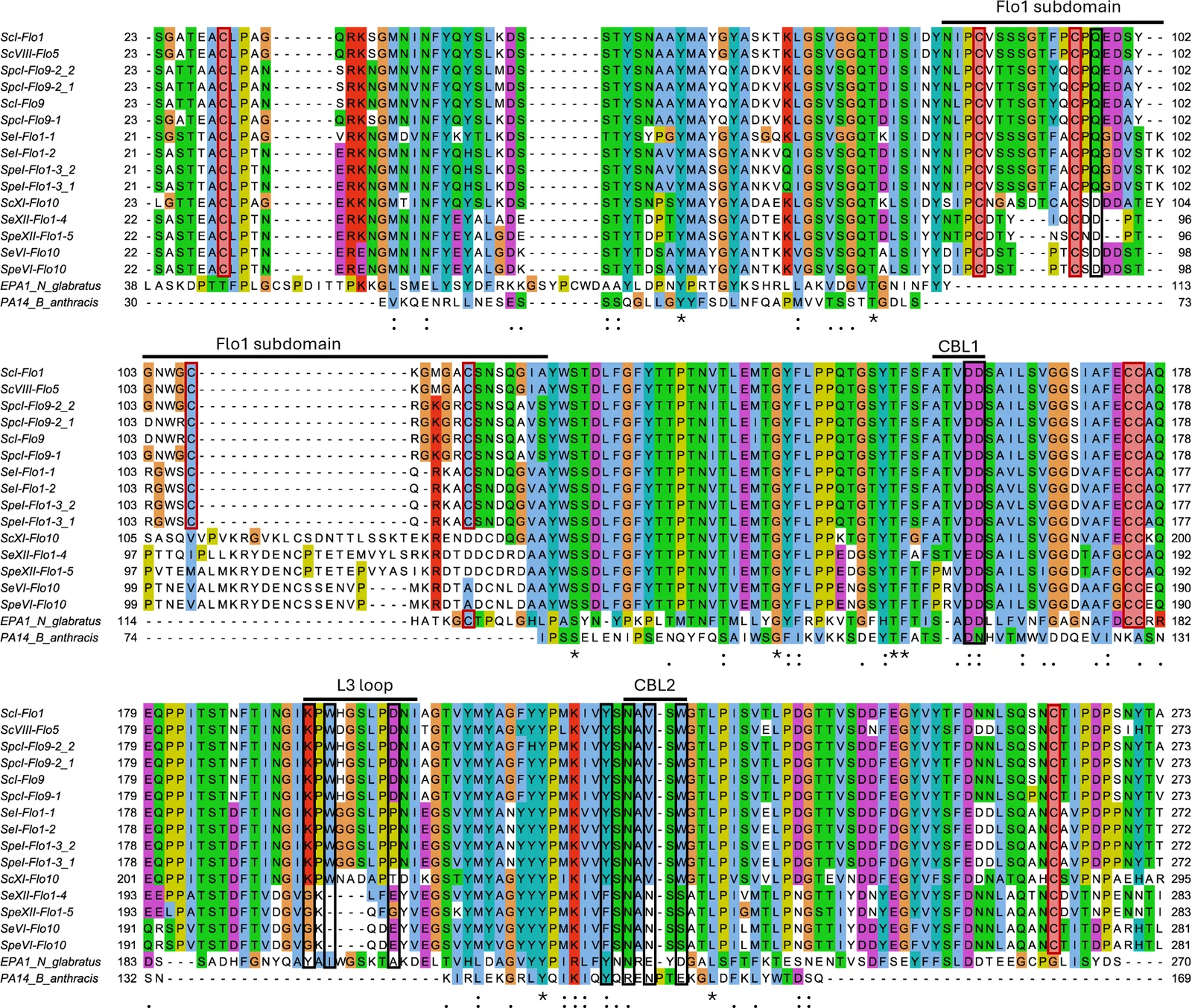
Multiple sequence alignment of the N-terminal carbohydrate PA14-binding site of flocculins from *S. cerevisiae* CEN.PK113-7D, *S. eubayanus* CDFM21L.1, and *S. pastorianus* CBS 1483. The PA14 domain from Epa1 from *Nakaseomyces glabratus* and the original Protective Antigen from *Bacillus anthracis* were included as references. The PA14 domain is highly conserved across all flocculins. *Se*VI-Flo10, *Spe*VI-Flo10, *Se*XII-Flo1-4, *Spe*XII-Flo1-5, and ScXIFlo10 show distinct variations in key amino acid residues relevant for Ca^2+^ binding and carbohydrate recognition compared to the remaining Flo1-like flocculins. Sequence alignment was done using Clustal Omega (71) and visualized using Jalview (85).

The L3 loop was also conserved, except in flocculins encoded on CHRVI and CHRXII, which lacked several hallmark residues. The D202 residue, which influences L3 loop flexibility and thereby carbohydrate specificity (Fig. 2), was only conserved in flocculins belonging to box 1 (Fig. 1B). Variations in CBL2 (e.g., C248N, W250S) may likewise further influence Ca²^+^ and carbohydrate recognition (74, 84, 86). Overall, the PA14 domain is highly conserved across Flo proteins from both parental species and their hybrid, with *Sc*I-Flo10 and the CHRVI/CHRXII *S. eubayanus*-derived proteins representing notable outliers that may use alternative ligand-recognition mechanisms.

### Repeat analysis

The central domain of Flo proteins consists of tandem repeats that modulate binding strength and ligand specificity (74, 76, 77, 80, 82). We previously reported five distinct motifs in Flo1-like proteins from *S. cerevisiae* CEN.PK113-7D, whereas Flo10 and Flo11 contained four and three types, respectively, with Flo10 sharing one motif with the Flo1-like proteins (33).

Repeat type composition was determined for all Flo proteins from *S. pastorianus* CBS 1483 and *S. eubayanus* CDFM21L.1 (Table 3). All *Spc*-Flo1-like proteins (box 1; Fig. 1) contained the same repeat types as their *S. cerevisiae* counterparts. Flo proteins from *S. eubayanus* contained Flo1 repeat types 1, 3, and 5 but lacked types 2 and 4, except *Spe*XII-Flo1-5 and *Se*XII-Flo1-4, which possessed Flo1 repeat types 1, 3, 4, and 5, plus an additional motif resembling an extended type 5 repeat. *Spc*I-Flo9-1, *Spc*I-Flo9-2, *Spe*I-Flo1-3, and *Spe*XII-Flo1-5 all contain a high number of repeat type 5, differing starkly from the *S. cerevisiae* proteins. These findings indicate that, while the overall repeat organization of Flo proteins is conserved between *Saccharomyces cerevisiae* and *eubayanus*, specific repeat variants have evolved.

### Flo12 is a hypothetical protein with similar structural organization as yeast adhesins

One protein, Flo12, was particularly distinct, sharing very low sequence identity with other flocculins (Fig. 1). A BLASTP analysis identified only uncharacterized proteins in other closely related *Saccharomyces* species, showing little similarity to any other known protein. Although Flo12 contained multiple internal repeats and a predicted C-terminal GPI anchor site, it notably lacks the characteristic N-terminal PA14 domain. Instead, it harbored a hyphally regulated cell wall protein N-terminal domain (Hyphal_reg_CWP, Pfam: PF11765) (Table 3), previously identified in *Candida albicans* adhesins involved in cell wall organization and host–fungus interactions (87, 88), and the cell wall protein Haze Protection Factor 1 (*HPF1*) and its homolog *HZY1* in *S. cerevisiae* (89–91). A Phyre2.2 (92) search revealed 85% structural similarity with 100% confidence to Adhesin-like wall protein 1 (Awp1) α-domain from *Candida glabrata*, while a protein sequence alignment of the two domains only showed 24.9% sequence similarity. Notably, the main tandem repeating motif in the central domain is distinct from the other flocculins, consisting of a serine/threonine-rich 16 amino acid sequence (Table S4). Downstream of the central domain region lies another novel repeating motif, followed by single Flo1 type 1 and type 3 repeats. The function of Flo12 is unknown, but it may contribute to the complex flocculation phenotype of *S. pastorianus*.

### Structural models highlight similarities and variations among flocculation proteins

Sequence alignment revealed substantial conservation within the PA14-binding domain, with limited yet potentially functionally relevant differences in key regions (Fig. 2). To explore the structural consequences of these variations, biomolecular structure predictions were simulated using Alphafold 3 (66) (Fig. 3). The predicted models displayed the canonical β-sandwich fold characteristic of the PA14 domains (74, 77, 79, 93) and Ca^2+^ coordination by conserved residues in CBL1 and CBL2. Structural alignment using US-align (94) showed high structural conservation across all PA14 domains (Fig. S2). Deviations in structure stem largely from the sequence variations observed in the Flo1 subdomain and L3 loop (Fig. 2), which may impact carbohydrate binding specificity (36, 74, 86). All PA14 domain-containing Flo proteins were predicted to bind mannose-α1,2-mannose-α1,2-mannose, a proxy of the D1 arm of N- and O-linked mannan chains of glycosylated proteins on the yeast surface.

**Fig 3:**
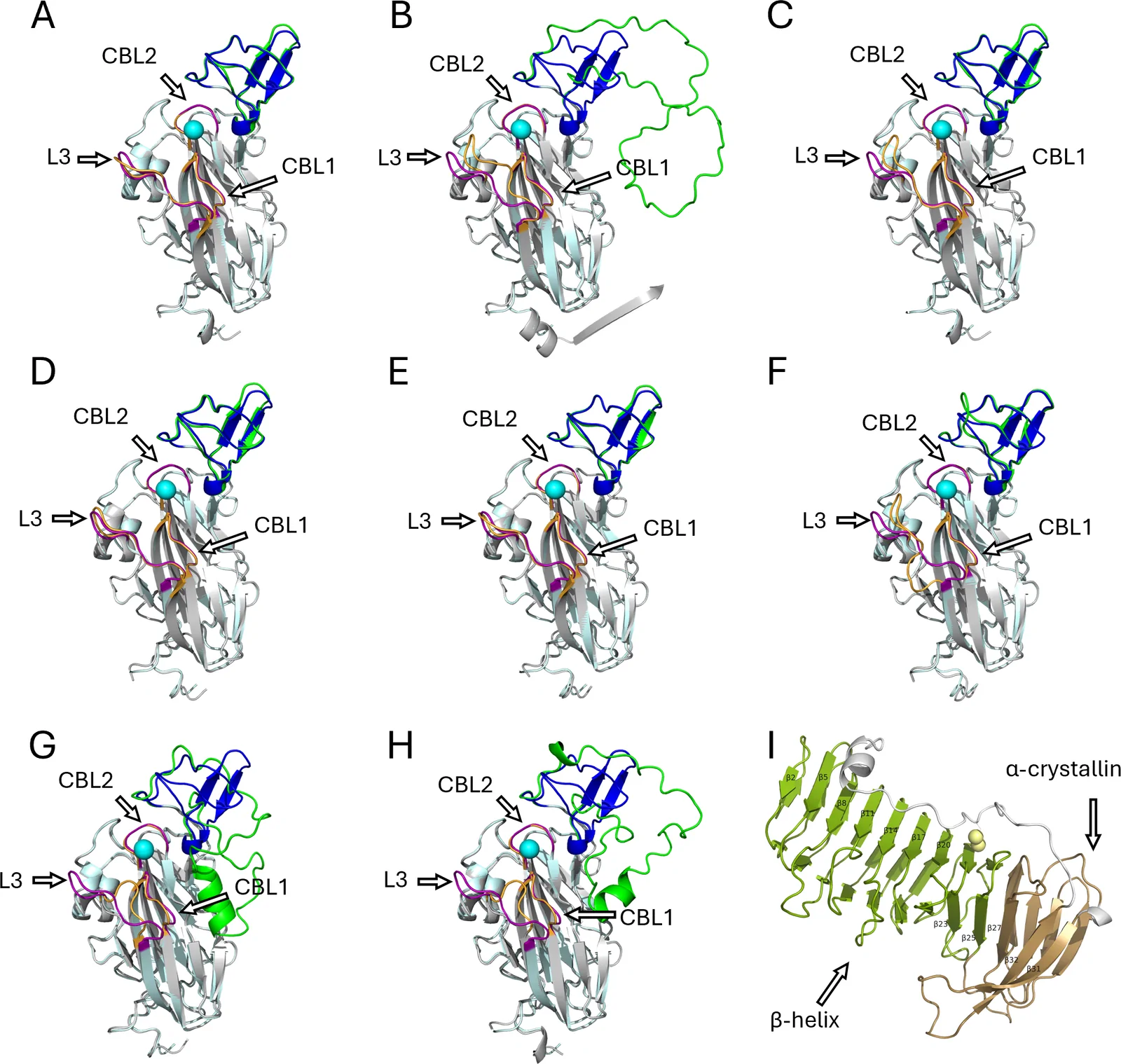
Alignment of predicted structures of the PA14 domains of flocculins (grey) with ScI-Flo1 (light blue) and structure of the Hyphal_reg_CWP domain of Flo12. The Flo1 subdomain is indicated in blue and green for ScI-Flo1 and the aligned flocculin, respectively. The relevant loops are indicated in purple and orange for ScI-Flo1 and the aligned flocculin, respectively. Ca^2+^ is indicated in cyan. (A) ScI-Flo9. (B) ScXI-Flo10. (C) SpcI-Flo9-1. (D) SpcI-Flo9-2_1. (E) SpcI-Flo9-2_2. (F) SpeI-Flo1-3. (G) SpeVI-Flo10. (H) SpeXII-Flo1-5. (I) The predicted structure of the Hyphal_reg_CWP domain in Flo12 strongly resembles *Candida* adhesins, displaying the characteristic β-helix and α-crystallin structures.

The Hyphal_reg_CWP domain of Flo12 was predicted to comprise a right-handed β-helix consisting of nine rings of three β-strands forming three stacked β-sheets in parallel, followed by an α-crystalline-like fold (Fig. 3I). A disulfide bond was predicted to bind the C-terminal section to the β-helix. This structure is characteristic of fungal adhesins such as the virulence factors, adhesin-like wall proteins (Awp) and Hyr/Iff-like (Hil) proteins, involved in adherence to host endo- and epithelial cells, and mediating biofilm formation in *Candida* spp*.* (87, 88). Based on these characteristics, Flo12 is likely involved in an adhesion phenotype.

### Functional assessment of *S. pastorianus* CBS 1483 flocculation genes using optogenetic control in a *FLO*-null strain background

To assess the functionality of the identified *FLO* genes, each of the eight full-length *FLO* genes, excluding *FLO11* (Table 3), was inserted into the optogenetic OptoQ-AMP5 circuit and characterized following a previously described workflow (33). As a benchmarking reference, *S. pastorianus* CBS 1483 cells harvested in the stationary phase exhibited a flocculation coefficient of 91.4% ± 1.2%, while CEN.PK113-7D and IMX2897 (*floΔ*) showed a flocculation coefficient of 7.2% ± 2.6% and 1.9% ± 4.2%, respectively (Fig. 4A). The OptoQ-AMP5 strains were grown in the dark and induced with light for 2 h, after which flocculation coefficients were determined. In the dark conditions, the flocculation coefficients ranged from −3.1% ± 5.7% to 26.1% ± 6.6%. After 2 h of induction, IMX3012 (*Spc*I-*FLO9*-1) and IMX3101 (*Spc*I-*FLO9-*2_1) were the only two strains showing a significant increase in flocculation coefficient, reaching 73.6% ± 8.8% and 65.7% ± 12.6%, respectively. However, these values remained lower than the coefficient of flocculation exhibited by IMX2912, which expresses the *S. cerevisiae Sc*I-*FLO1* (33), and reached 96.6% ± 2.3%. Since all strains have the same transcriptional regulation, differences in flocculation strength are likely attributable to intrinsic differences in flocculin activity/affinity. Although flocculin expression was not quantified, the sequence of each expression cassette was verified and revealed no mutations or structural variations. Therefore, these differences are unlikely to result from defects in the expression constructs and may instead be mitigated by increasing flocculin surface expression. To test this hypothesis, we modified the assay by extending the light induction period to 24 h. Under these conditions, IMX3012 (*Spc*I-*FLO9*-1) and IMX3101 (*Spc*I-*FLO9*-2_1) reached flocculation of 98.1% ± 0.1% and 94.6% ± 0.2%, respectively, values comparable to IMX2912 (*Sc*I*-FLO1*) after only 2 h of induction. Under the extended induction regime, IMX3013 (*Spe*VI-*FLO12*) and IMX3102 (*Spc*I-*FLO9*-2_2) also showed substantial increases, reaching 57.9% ± 9.8% and 80.1% ± 3.5%, respectively. IMX3104 also showed a significant increase in flocculation coefficient after 24 h, reaching 28.5% ± 0.8%, but did not show any measurable increase in floc formation. None of the remaining strains exhibited a significant increase in flocculation coefficient, and microscopy confirmed the absence of visible flocs. Among the flocculating strains, floc sizes were generally smaller than those of IMX2912 (*Sc*I-*FLO1*), which exhibited an interquartile range (IQR) of 693–9,363 μm^2^ (Fig. 4B; Fig. S4). After 24 h of induction, IMX3102 (*Spc*I-*FLO9*-2_2) formed the largest flocs with an IQR of 144–1,070 μm^2^, consistent with its flocculation coefficient. IMX3101 (*Spe*VI-*FLO9*-2_1) showed smaller floc sizes with an IQR of 150–464 μm^2^. Notably, although IMX3013 (*Spe*VI-*FLO12*) displayed an increase in flocculation coefficient, the geometric mean of the floc size was not significantly different from IMX2897 (*floΔ*). However, IMX3013 showed an IQR of 107.6–202.5 μm^2^ after 24 h of light induction, showing a slight increase in larger particles appearing more frequently (Fig. 4B; Fig. S4). This minor increase may be enough to trigger sedimentation. Visual inspection of the flocculation assay tube revealed very small, sand-like particles. Together, these results highlight the diversity of flocculation phenotype among *S. pastorianus FLO* genes and suggest substantial variation in aggregation potential of the encoded flocculins.

**Fig 4:**
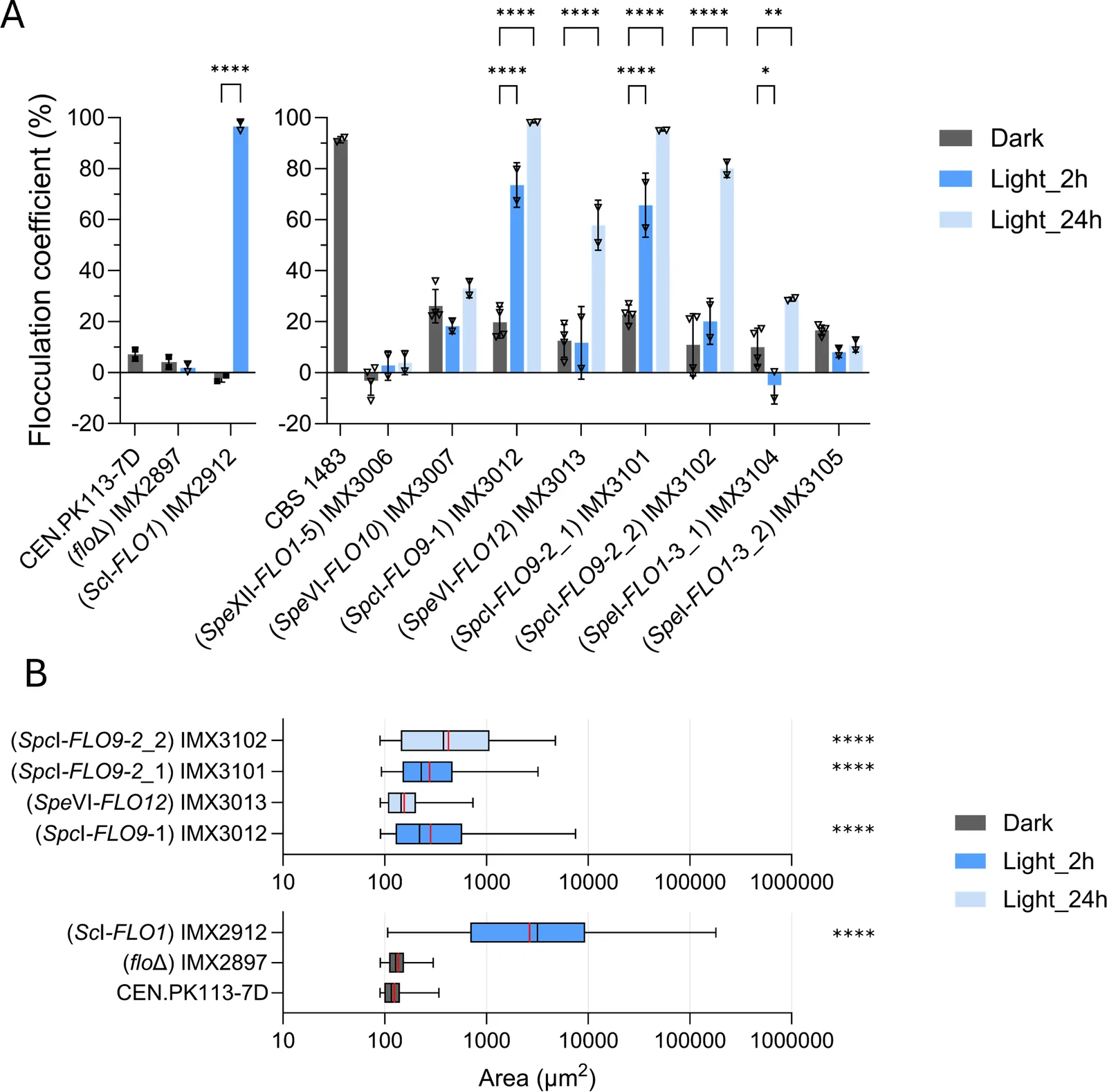
(**A**) Flocculation coefficients of CEN.PK113-7D, CBS 1483, IMX2897 (*floΔ*), and optogenetics strains expressing *Sc*I-*FLO1* and CBS 1483 *FLO* genes sampled after 2 and 24 h of growth under light and dark conditions. CBS 1483 was sampled at the stationary phase. For each condition, strains were grown in duplicate cultures, and triplicate technical replicates were performed to determine the flocculation coefficient for each culture. IMX3012 (*Spc*I*-FLO9*-1) and IMX3101 (*Spc*I*-FLO9*-2_1) showed a significant increase in flocculation coefficient after 2 h of induction, while IMX3013 (*Spe*VI-*FLO12*) and IMX3102 (*Spc*I*-FLO9*-2_2) only showed a significant increase after 24 h of induction (*P < 0.05, **P < 0.01, ****P < 0.0001). IMX3104 (*Se*I-*FLO1*-3_1) showed a minor significant increase after 24 h of induction, but no significant increase in floc formation was observed. All other strains show no significant increase relative to dark conditions. (**B**) Floc size distribution based on the area of cell aggregates imaged at 200× magnification using microscopy. The floc sizes of IMX2912 (*Sc*I-*FLO1*) show a larger range and geometric mean relative to the flocs induced by CBS 1483 *FLO* genes. All strains form significantly larger floc sizes relative to IMX2897 (*floΔ*) (****P < 0.0001), with the exception of IMX3013 (*Spe*VI-*FLO12*), despite showing a moderate flocculation coefficient. All particles smaller than 90 μm^2^ were filtered out to remove aggregates smaller than two aggregated budding cells. Statistical significance was calculated using a lognormal one-way ANOVA test. Each box represents the 25th to 75th percentiles, with the whiskers indicating the minimum and maximum values. The black line represents the median, while the red line represents the geometric mean of the samples. The number of cell aggregates analyzed per strain ranged from 59 to 147. The data for CEN.PK113-7D, IMX2897 (*floΔ*), and IMX2912 (*Sc*I-*FLO1*) in panels A and B were obtained from our previous study (33).

### Sugar inhibition phenotypes of CBS 1483 flocculins

*S. pastorianus* strains are known to exhibit a NewFlo flocculation phenotype, showing sensitivity to various sugars, e.g., mannose, maltose, glucose, and sucrose, low pH conditions, and proteinases (40, 77, 86). To identify which of the flocculation genes are involved in flocculation in CBS 1483, the sensitivity of the functional flocculins *Spc*I*-*Flo9-1, *Spc*I*-*Flo9-2_1, and *Spc*I-Flo9-2_2 to various fermentable sugars found in wort was determined as previously described (33) (Fig. 5A). All the sugars tested completely abolished flocculation in CBS 1483. Notably, we demonstrated the sensitivity of *S. pastorianus* to fructose and maltotriose. IMX3012 (*Spc*I-*FLO9*-1) showed sensitivity mainly to mannose, sucrose, and, to a lesser degree, fructose, maltose, and maltotriose. Additionally, IMX3101 (*Spe*VI-*FLO9*-2_1) and IMX3102 (*Spc*I-*FLO9*-2_2) showed different inhibition characteristics. IMX3101 (*Spe*VI-*FLO9*-2_1) was sensitive to mannose, sucrose, maltose, and, to a lesser degree, maltotriose, while IMX3102 (*Spc*I-*FLO9*-2_2) showed an additional sensitivity to fructose and glucose. Remarkably, none of the tested strains exhibited the stark inhibition by all the sugars as was observed for CBS 1483.

**Fig 5:**
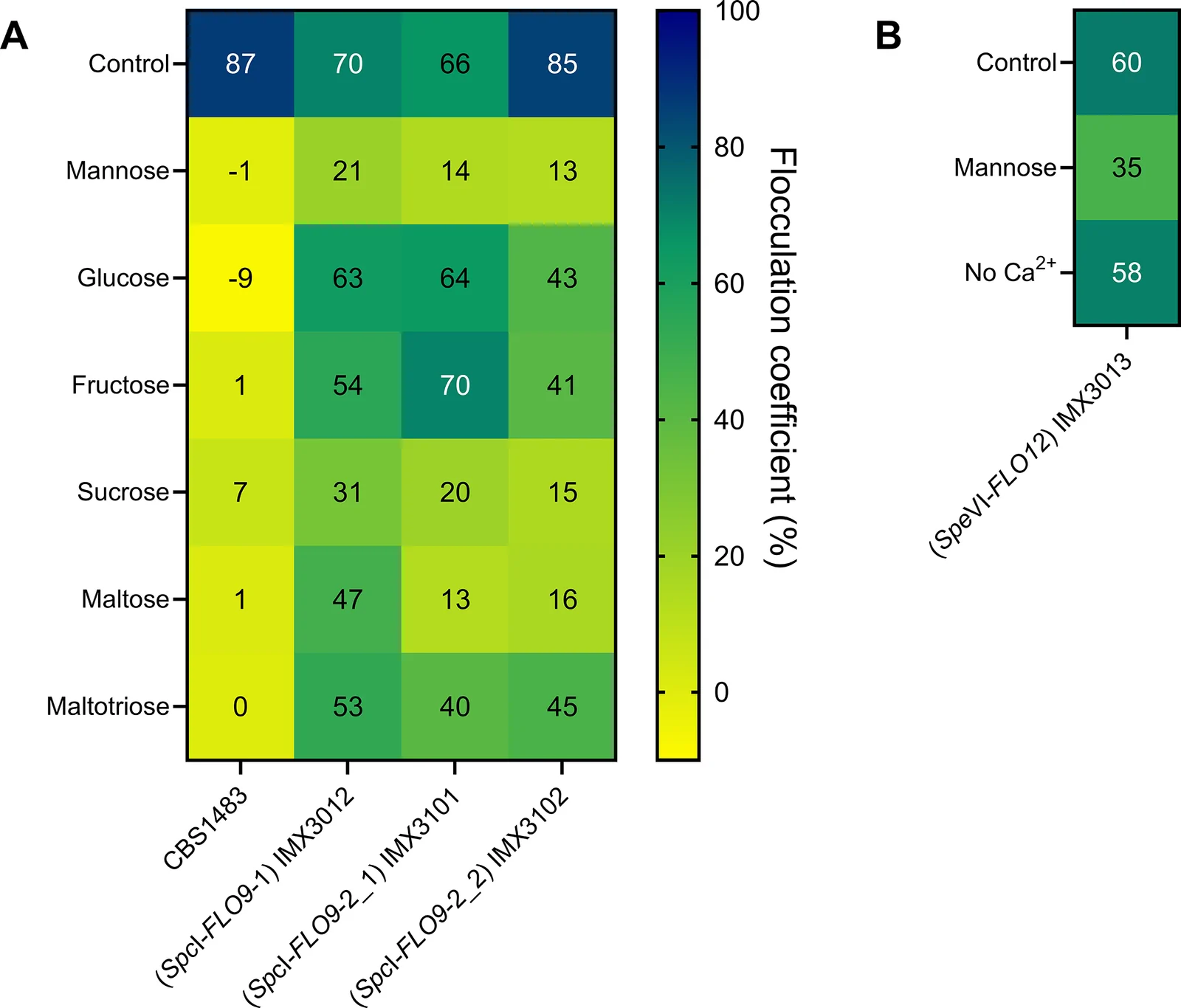
Sugar inhibition profiles of (**A**) CBS 1483, IMX3012 (*Spc*I*-FLO9*-1), IMX3101 (*Spc*I*-FLO9-*2_1), IMX3102 (*Spc*I*-FLO9-*2_2), and (**B**) mannose and Ca^2+^ sensitivity of IMX3013 (*Spe*VI-*FLO12*). The optogenetic strains were induced until measurable flocculation occurred: 2 h for IMX3012 (*Spc*I*-FLO9*-1) and IMX3101 (*Spc*I*-FLO9-*2_1), and 24 h for IMX3102 (*Spc*I*-FLO9-*2_2) and IMX3013 (*Spe*VI-*FLO12*). (**A**) CBS 1483 showed a strong sensitivity to all the tested sugars. IMX3012 (*Spc*I*-FLO9*-1), IMX3101 (*Spc*I*-FLO9-*2_1), and IMX3102 (*Spc*I*-FLO9-*2_2) all exhibited similar sugar sensitivity profiles, showing strong sensitivity to mannose, with weak to moderate sensitivity to the remaining sugars. (**B**) IMX3013 (*Spe*VI-*FLO12*) showed Ca^2+^-independent flocculation that was moderately sensitive to mannose.

As IMX3013 (*Spe*VI-*FLO12*) exhibited flocculation behavior, we tested it for Ca^2+^ requirement and mannose sensitivity (Fig. 5B). There was no significant reduction in flocculation coefficient in the samples without Ca^2+^ supplementation (58% ± 6.4%) relative to the control (60% ± 13.2%). Interestingly, 1 M mannose caused a reduction of 42% in flocculation coefficient relative to the control, suggesting sugars may interact with the Hyphal_reg_CWP domain.

### CBS 1483 flocculins exhibit varying impact on cell surface hydrophobicity

Flocculins are known to contribute to cell wall hydrophobicity to varying degrees (33, 38, 95, 96). Using the previously described dodecane partitioning assay, the effect of the CBS 1483 flocculins on the cell surface hydrophobicity was determined (Fig. 6). Flocculating CBS 1483 cells grown to the stationary phase displayed a hydrophobicity percentage of 46.5% ± 7.3%, while the non-flocculating CEN.PK113-7D showed a hydrophobicity percentage of 18.3% + 11.0%. CBS 1483 was comparable to the strongly flocculating IMX2912 (*Sc*I-*FLO1*), which exhibited a hydrophobicity percentage of 51.3% ± 3.5%. Notably, IMX3006 (*Spe*XII-*FLO1-*5) and IMX3007 (*Spe*VI-F*LO10*) showed a strong increase in cell surface hydrophobicity, reaching values of 76.5% ± 2.3% and 55.0% ± 3.3%, respectively, despite their encoded Flo proteins failing to initiate flocculation (Fig. 6A). In contrast, the flocculating strains IMX3012 (*Spc*I-*FLO9-*1), IMX3101 (*Spc*I-*FLO9*-2_1), and IMX3102 (*Spc*I-*FLO9*-2_2) did not show a significant increase in cell surface hydrophobicity relative to dark conditions. The strain IMX3013 (*Spe*VI-*FLO12*), which expresses a novel Flo protein lacking a PA14 lectin-binding domain, exhibited a strong increase in hydrophobicity, reaching 75.8% ± 1.0%. IMX3104 (*SpeI-FLO1*-3_1) and IMX3105 (*Spe*I-*FLO1*-3_2) showed no significant increase in cell surface hydrophobicity. Together, these data illustrate that increases in cell surface hydrophobicity do not necessarily coincide with cellular aggregation, highlighting distinct mechanistic contributions of different flocculins.

**Fig 6:**
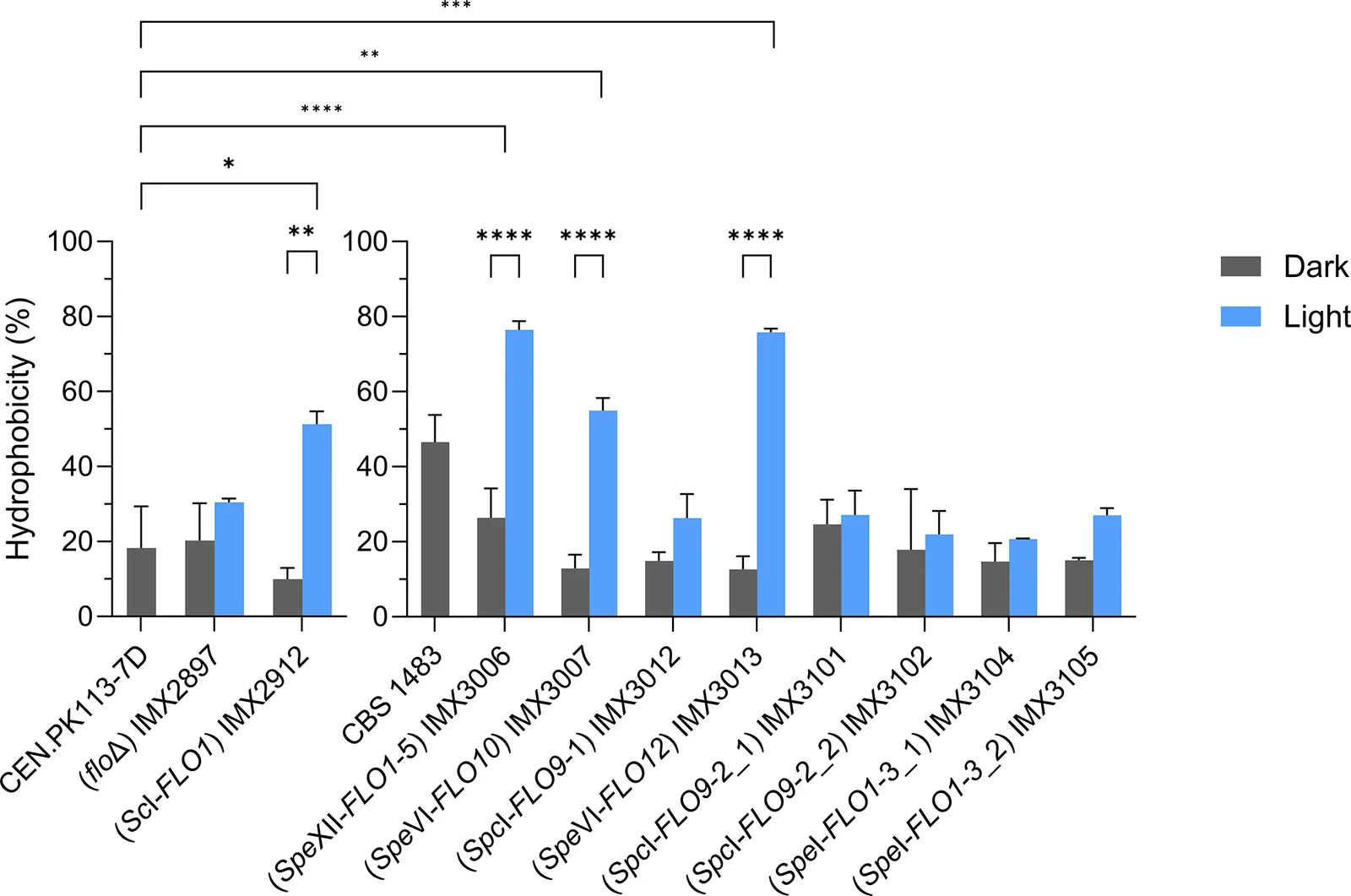
Determination of cell surface hydrophobicity of CEN.PK113-7D, CBS 1483, and optogenetic strains expressing *Sc*I-*FLO1* (33) and the identified CBS 1483 *FLO* genes grown under light and dark conditions. The hydrophobicity percentage is calculated by the ratio of cells partitioning between an aqueous phase and a dodecane organic phase. All bars represent average values of experiments using biological duplicates and measured in technical duplicates. The CEN.PK113-7D bar represents the average of all control experiments (33). IMX3006 (*Spe*XII-*FLO1*-5), IMX3007 (*Spe*VI-*FLO10*), and IMX3013 (*Spe*VI-*FLO12*) showed a significant increase in hydrophobicity in light conditions relative to dark conditions (*P < 0.05, **P < 0.01, ***P < 0.001, ****P < 0.0001). CBS 1483 and IMX2912 (*Sc*I-*FLO1* light) showed a similar increase in hydrophobicity. All strains grown in darkness showed no significant difference in hydrophobicity relative to CEN.PK113-7D. Error bars represent the standard deviation. Statistical significance was calculated using multiple unpaired two-sided *t*-tests. *P*-values were corrected for multiple comparisons using the Holm-Šidák method. The data for CEN.PK113-7D and IMX2912 (*Sc*I-*FLO1*) were obtained from our previous study (33).

### *FLO* gene expression analysis in 17° Plato wort fermentation of CBS 1483 in E.B.C. tall tubes

To identify which of the flocculin genes contribute to the strong flocculation phenotype observed in group II strain CBS 1483, a transcriptome analysis was performed by sampling cells at various time points during a 17° Plato wort fermentation conducted in E.B.C. tall tubes for 15 days (Fig. 7). The *S. pastorianus* CBS 1483 strain started to flocculate around day 5 (Fig. 7A). Samples were collected on days 1, 2, 5, 6, 9, 12, and 15 for transcriptome analysis. After 5 days of fermentation, the cell counts peaked and subsequently declined, showing a 72% decrease by day 6 and complete sedimentation of the biomass by day 8 (Fig. 7A). The onset of sedimentation coincided with the depletion of most fermentable sugars after 6 days (Fig. 7A and B). At the end of fermentation, residual concentrations of maltose and maltotriose remained at 2.4 g·L^−1^ and 13.6 g·L^−1^, respectively (Fig. 7B), and a final gravity of 4.1 was reached (Fig. 7A). Out of the nine full-length *FLO* genes (including allelic variants, and excluding *FLO11* and the truncated *FLO* genes) detected on CBS 1483 genome (Table 3), eight could form a detectable transcript (Fig. 7C), namely *Spc*I-*FLO9*-1, *Spc*I-*FLO9*-2_1, *Spc*I-*FLO9*-2_2, *Spe*I*-FLO1*-3_1, *Spe*VI-*FLO12*, *Spe*VI-*FLO10*, and *Spe*XII-*FLO1*-5 (Table 3).

**Fig 7:**
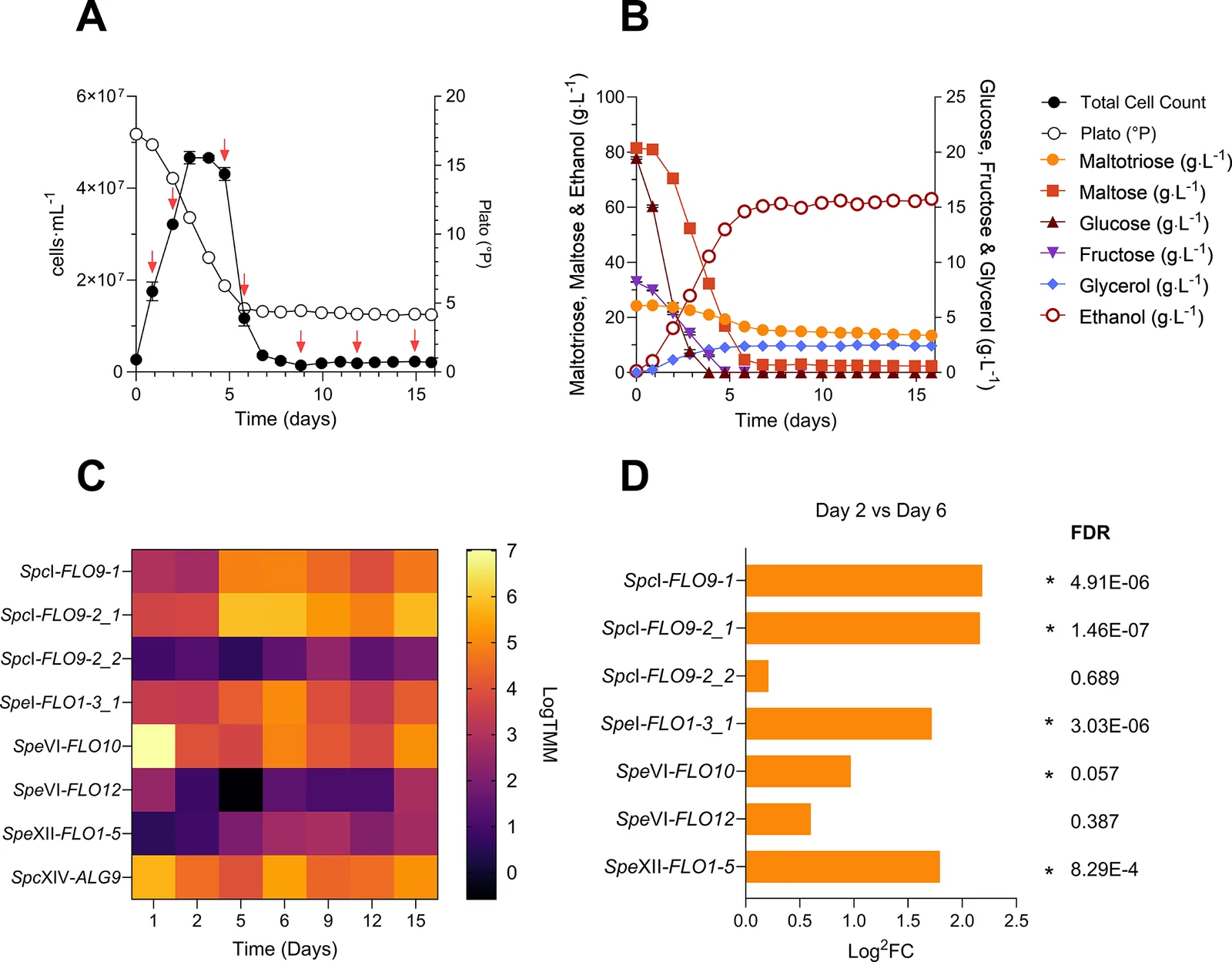
Transcriptomic analysis of *FLO* gene expression in CBS 1483 during a duplicate 17°P wort fermentation at 12°C in E.B.C. tall tube fermenters. (**A**) Cell count (cells·mL^−1^) of CBS 1483 and wort density (°P) during the 15-day-long fermentation. Red arrows indicate sampling timepoints for transcriptomic analysis. The cell count drops dramatically after 5 days, indicating flocculation, and coincides with attenuation of the wort. (**B**) Fermentable sugars, glycerol, and ethanol concentrations during the CBS 1483 fermentation. (**C**) Transcription profile of detected *FLO* genes during the CBS 1483 fermentation expressed as TMM values. (**D**) Fold changes of *FLO* genes between and day 6 relative to day 2.

Overall, *FLO* genes’ expression levels were relatively low, with a subset showing a dynamic expression pattern coinciding with the onset of flocculation and sedimentation at day 5 (Fig. 7C; Table S5). Expressed in reads per kilobase of transcript per million reads mapped (rpkm), *FLO* gene transcripts never exceeded 38.5 ± 0.92 rpkm. Although slightly higher than the median transcript level across all samples (23.36 rpkm), the *FLO* transcripts were at least an order of magnitude lower than maltose transporter genes *Spc*II-*MAL31,* which reached 743.9 ± 68.42 rpkm at day 6, or glycolytic genes *Spc*IV-*TPI1* and *Spe*III-*c*III-*PGK1*, which reached expression levels of 8013.3 ± 184.38 and 5828.0 ± 33.74 rpkm, respectively, on the first day. After *Spe*VI-*FLO10*, *Sp*cI-*FLO9*-2_1 showed the highest rpkm value at 19.3 ± 9.31 on day 15, followed by 19.0 ± 0.90 and 18.7 ± 2.90 on days 5 and 6, respectively. We monitored more specifically the changes between day 2 and day 6. *Spc*I-*FLO9*-1 and *Spc*I-*FLO9*-2_1 both exhibited a 4.5-fold increase in transcript levels on day 6 relative to day 2 (Fig. 7D). Notably, *Spc*I-*FLO9*-2_2 showed very low expression relative to *Spc*I-*FLO9*-2_1, indicating preferential allelic expression. *Spe*I-*FLO1*-3 and *Spe*XII-*FLO1*-5 showed a 3.2- and 3.5-fold increase on day 6 relative to day 2. Notably, *Spe*VI-*FLO10* shows the highest expression with 38.5 ± 0.92 rpkm on day 1, while the cells are still in free suspension. After 6 days, transcript levels of all *FLO* genes declined. An increase was also observed on day 15, reaching expression levels comparable to those reached at day 5, which corresponded to the planktonic cellular fraction that still had to flocculate. These results suggest that *Spc*I-*FLO9*-1, *Spc*I-*FLO9*-2, followed by *Spe*I-*FLO1*-3 and potentially *Spe*VI-*FLO10,* are likely the main contributors to the pronounced flocculation phenotype observed in CBS 1483. The novel *Spe*VI-*FLO12* gene and *Spe*XII-*FLO1*-5 exhibited very low levels of expression and are likely not involved in the observed flocculation phenotypes.

### Knockout of *Spc*I-*FLO9*-1 and *Spc*I-*FLO9*-2 did not abolish flocculation in CBS 1483 but improved overall brewing performance

Data integration revealed that among the flocculin genes *Spc*I-*FLO9*-1, *Spc*I-*FLO9*-2_1, *Spc*I-*FLO9*-2_2, and *Spe*VI-*FLO12*, all previously associated with initiation of a flocculation phenotype, only *Spc*I-*FLO9*-1 and *Spc*I-*FLO9*-2_1 exhibited detectable transcriptional activity (Fig. 7). To determine whether these genes were the sole genetic determinants of flocculation in CBS 1483, a double knockout strain lacking both *Spc*I-*FLO9*-1 and *Spc*I-*FLO9*-2 was constructed, yielding strain IMX3185 (*SpcI-flo9*-1Δ::NatNT2 *Spc*I-*flo9*-2Δ::KanMX). IMX3185 and CBS 1483 were then cultivated in 17° Plato wort in E.B.C. tall tubes to test the effects of the knockouts under brewing-relevant conditions (Fig. 8). As expected, the parental strain exhibited flocculation onset at day 5, followed by a progressive decline in planktonic biomass until day 10. Surprisingly, IMX3185 also exhibited flocculation, with a profile similar to that of CBS 1483, albeit delayed by approximately 24–30 h. By day 10, planktonic cells were largely sedimented in both strains. Consistent with this delayed flocculation, IMX3185 showed slightly improved attenuation. The mutant strain reached a final gravity of 4.25°P, compared to 4.6°P for CBS 1483, which correlated with a lower residual maltose concentration at the end of fermentation (4.7 ± 0.4 g L⁻¹ versus 6.81 ± 0.5 g L⁻¹, respectively). Overall, these results suggest that compensatory mechanisms are likely activated to counteract the absence of *Spc*I-*FLO9*-1 and *Spc*I-*FLO9*-2, thereby partially restoring the flocculation phenotype under wort fermentation conditions.

**Fig 8:**
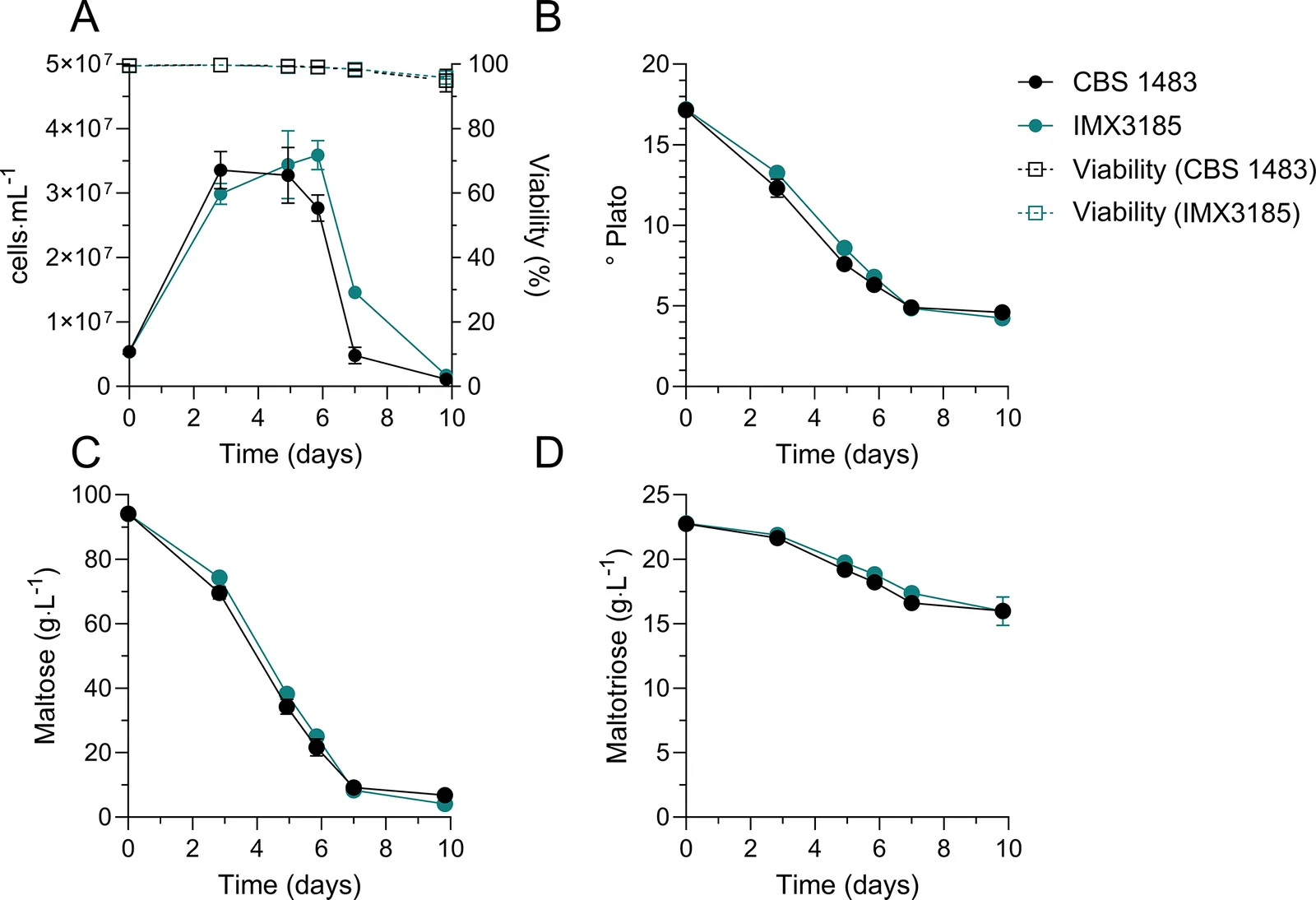
Measured cell count (cells·mL^−1^) (**A**), wort density (°P) (**B**), and maltose (**C**) and maltotriose concentrations (**D**) of CBS 1483 and the double knock-out strain IMX3185 (*Spc*I-*FLO9*-1Δ::NatNT2 *Spc*I-*FLO9*-2Δ::KanMX) during a 17°P wort fermentation at 12°C in E.B.C. tall tubes.

## DISCUSSION

Flocculation in *Saccharomyces* species has traditionally been framed as a phenotype driven by highly potent, dominant flocculins such as Flo1, whose overexpression is sufficient to induce rapid and extensive cell aggregation (1, 30, 97). However, our systematic analysis of the *FLO* repertoire of *Saccharomyces pastorianus* CBS 1483 challenges this classical single-driver model. Instead, our data support a distributed architecture in which moderately active adhesins, expressed at relatively low levels, collectively shape the characteristic sedimentation behavior of lager yeast.

Optogenetic characterization in a *FLO*-null background (33) demonstrated that *Spc*I-Flo9-1 and *Spc*I-Flo9-2_1 possess clear aggregation capacity. However, their intrinsic performance remains below that of canonical *Sc*I-Flo1 under identical transcriptional control. The most likely factor underlying this difference is protein architecture and, more specifically, the number of tandem repeats. Previous studies have established that flocculin activity is directly related to repeat number, with a lower number of repeats generally resulting in reduced flocculation capacity (98). Consistent with this observation, the *Sc*I-FloI from CEN.PK113-7D is 1,675 amino acids long and contains 20 type 1 repeats, whereas the *Spc*I-Flo9-1 and *Spc*I-Flo9-2_1 proteins from *S. pastorianus* CBS 1483 are only 1,043 and 869 amino acids long, and contain six and one type I repeat, respectively. These structural differences likely account for the observed variation in aggregation activity. More intriguingly, they suggest that despite the importance of flocculation in the brewing process, the domestication of lager yeast did not necessarily favor evolution or retention of the most active flocculins. This observation may indicate that an optimal rather than a maximal level of flocculation was selected during the adaptation of *S. pastorianus* to industrial brewing environments. Therefore, only prolonged induction allowed these proteins to reach comparable flocculation coefficients, indicating lower intrinsic binding strength. *Spc*I-Flo1-2_2 and the structurally distinct *Spe*VI-Flo12 showed limited or delayed aggregation potential. Furthermore, several flocculins substantially increased cell surface hydrophobicity without triggering macroscopic flocculation, highlighting that adhesive surface properties and intercellular aggregation are not equivalent phenomena (74, 84).

It is important to note that CBS 1483 and the *FLO*-null platform strain belong to different species, which may influence protein folding, glycosylation, and flocculin function at the cell surface. Differences in cell wall composition and surface architecture may therefore contribute to the observed flocculation phenotypes. Comparative studies have revealed variation in mannoprotein content, cell wall elasticity, polysaccharide structure, and carbohydrate composition among yeast strains (99–101). In addition, differences in the spatial organization of flocculins may influence flocculation behavior (84). However, the contribution of these factors remains poorly understood. Comparative analyses across different strain backgrounds may help identify additional mechanisms underlying *FLO*-mediated flocculation.

Transcriptomic analysis during 17°P wort fermentation revealed that *FLO* genes are expressed at modest levels relative to highly expressed metabolic genes. Even at peak induction around day 5, coinciding with the onset of sedimentation, transcript levels remained low. No single *FLO* gene exhibited dominant transcription that would indicate a master flocculin controlling the phenotype. Instead, flocculation onset correlated with moderate induction of multiple adhesins, most notably *Spc*I-*FLO9-*1 and *Spc*I-*FLO9-*2_1, alongside contributions from *Spe*I-*FLO1-*3 and potentially *Spe*VI-*FLO10*. This coordinated yet restrained expression pattern is consistent with a network-based model rather than a single-gene determinant (38).

The double deletion of *Spc*I-*FLO9-*1 and *Spc*I-*FLO9-*2 provides further support for this distributed control mechanism. The deletions did not eliminate sedimentation in the E.B.C. tube wort fermentations but merely delayed it by approximately 24–30 h. Ultimately, the mutant sedimented efficiently and even showed improved attenuation due to prolonged sugar consumption. These observations strongly suggest activation of compensatory mechanisms within the *FLO* network.

Such compensation is not unprecedented in yeast biology. *Saccharomyces cerevisiae* possesses numerous paralogous gene families that exhibit transcriptional compensation upon deletion of a dominant member. A classic example is the pyruvate decarboxylase system: deletion of *PDC1*, which encodes the major isoenzyme, triggers strong upregulation of *PDC5*, restoring fermentative flux through autoregulatory mechanisms (102–104). Similarly, deletion of *HXK2*, the dominant hexokinase under glucose-repressed conditions, induces *HXK1* expression (105). Enolase paralogs exhibit comparable behavior, with *ENO1* upregulated upon *ENO2* deletion (106). Compensation also occurs in ribosomal protein genes (e.g., *RPL1A*/*RPL1B*) (107), glucose transporters (*HXT* family via Snf3/Rgt2 signaling) (108), glycerol-3-phosphate dehydrogenases (*GPD1*/*GPD2*) (109), and cytosolic malate dehydrogenases (*MDH2*/*MDH3*) (110).

These examples illustrate a recurring principle in yeast genetics: functionally overlapping paralogs can buffer perturbations through transcriptional or physiological compensation, thereby maintaining system-level robustness. The *FLO* gene family, particularly in the hybrid genome of *S. pastorianus*, is ideally structured for such buffering. CBS 1483 contains multiple subgenome-derived *FLO* genes, allelic variants with distinct repeat compositions, and structurally diverse adhesins such as Flo12. This redundancy creates a landscape in which loss of dominant contributors may be partially offset by increased relevance, whether transcriptional, post-transcriptional, or functional, of secondary flocculins.

One can argue that the selective pressure acting on flocculins differs fundamentally from that acting on metabolic isozymes. Loss of a metabolic isozyme can create an immediate fitness defect by impairing growth, whereas loss of a flocculin does not directly prevent cell proliferation. However, in the context of industrial lager brewing, flocculation is subject to indirect but persistent selection. The brewing process relies on serial re-pitching, whereby flocculated biomass is recovered at the end of fermentation and used to inoculate subsequent batches. Consequently, strains with impaired flocculation may be disadvantaged during repeated propagation cycles, providing a selective pressure to maintain sufficient aggregation capacity. In addition, *FLO* genes are characterized by extensive tandem-repeat regions that are prone to expansion and contraction. Changes in repeat number can substantially alter flocculin activity and therefore provide a mechanism through which aggregation capacity could be modulated. While we do not claim to have demonstrated a compensatory mechanism, we propose that the selective pressure imposed by industrial re-pitching, together with the structural plasticity of *FLO* genes, could facilitate the maintenance or restoration of flocculation capacity following the loss or reduced activity of individual flocculins, as observed in this study.

Although we did not experimentally validate whether alternative *FLO* genes were upregulated upon deletion of *Spc*I-*FLO9-*1 and *Spc*I-*FLO9-2,* compensation in yeast is not always mediated by large transcriptional shifts. In several paralog systems, modest changes in expression, altered metabolic flux, or changes in cellular context are sufficient to restore phenotype (111–113). In wort, environmental parameters such as ethanol accumulation, nutrient depletion (14), pH shifts (17), and Ca²^+^ availability may alter the effective binding affinity or cell-surface exposure of remaining adhesins (74). Thus, *Spe*I-Flo1-3 or *Spe*VI-Flo10, insufficient alone under laboratory conditions, may become functionally significant in the physiological context of fermentation.

Importantly, the compensatory phenotype observed here does not restore rapid or excessive flocculation, but rather produces a delayed yet orderly sedimentation profile. From an industrial standpoint, this is advantageous. Excessively strong flocculation can cause premature biomass removal, incomplete maltose and maltotriose consumption, and formation of off-flavors. Conversely, weak flocculation prolongs clarification and increases tank occupancy. The lager yeast phenotype appears finely tuned to initiate sedimentation only after substantial sugar depletion. The NewFlo-like sugar sensitivity of *Spc*I-Flo9 proteins ensures inhibition during early fermentation stages, while moderate expression and distributed activity prevent abrupt aggregation.

Lg*-FLO1* is often proposed as the primary determinant of NewFlo-type flocculation in lager yeast strains (40, 41, 43, 86, 97). However, its absence in CBS 1483, consistent with previous reports (46), demonstrates that Lg-*FLO1* is not universally required for this phenotype. In CBS 1483, the corresponding locus contains a recombination-derived *FLO*/*HZY1* (YIL169C) hybrid gene, with an additional recombination event on the *Spe*X*-c*X chromosome. Such rearrangements have been associated with loss of flocculation in *S. pastorianus*, indicating that Lg*-FLO1* is unlikely to contribute to flocculation in this strain.

Despite lacking a functional Lg-*FLO1*, CBS 1483 clearly displays a NewFlo phenotype. None of the identified *FLO* genes contain the KALAR/KVLAR motif previously linked to broad sugar recognition, suggesting that this motif is not essential for NewFlo-type flocculation. This is consistent with reports that *S. cerevisiae FLO9* and *FLO10* can confer NewFlo-like behavior despite lacking canonical sequence features (22, 33, 78), implying that alternative molecular mechanisms underlie sugar sensitivity in flocculins.

We therefore propose that the lager flocculation phenotype represents an emergent property of a moderately expressed adhesin network rather than dominance of the strongest flocculins known in *S. cerevisiae*. The hybrid genome of *S. pastorianus* may have facilitated this evolutionary outcome by providing a diverse repertoire of adhesins with graded binding capacities. Over centuries of domestication under cold, high-gravity conditions, strains exhibiting balanced sedimentation kinetics, neither too rapid nor too slow, would have been preferentially retained.

In this context, compensatory capacity becomes a feature rather than a flaw. The persistence of flocculation upon deletion of major *FLO* genes demonstrates robustness embedded within the adhesin network. Similar to central carbon metabolism or ribosome assembly systems, redundancy ensures stability under genetic or environmental perturbation. Such robustness likely contributes to the reliability and reproducibility demanded in industrial lager brewing. The assembly of *FLO* genes is particularly challenging when relying on Illumina short-read sequencing, which remains the most widely used approach for sequencing *Saccharomyces pastorianus* strains. Members of the *FLO* gene family share extensive sequence homology while also containing long internal arrays of tandem repeats. Because these highly similar and repetitive regions often exceed the length of individual Illumina reads, assembly algorithms frequently fail to unambiguously assign reads to specific loci or accurately resolve repeat copy numbers. Consequently, *FLO* genes are prone to fragmentation, repeat collapse, and misassembly in short-read-based genome assemblies, limiting the extension of flocculin analyses to additional *S. pastorianus* strains.

The adoption of long-read sequencing technologies, which generate reads capable of spanning entire repeat arrays and extending into unique flanking regions, should greatly improve the resolution of *FLO* loci. This would enable more accurate reconstruction of gene structure, repeat number, and copy number variation, thereby facilitating robust comparisons of flocculin repertoires across *S. pastorianus* strains. In conclusion, our findings support a revised model of flocculation control in *S. pastorianus*: rather than being driven by a single high-performance flocculin, sedimentation behavior emerges from the collective action of multiple moderately active adhesins whose expression and activity are finely modulated during fermentation. Compensatory interactions within this network buffer genetic perturbations and ensure that biomass depletion proceeds with kinetics optimally aligned to lager fermentation. This systems-level perspective provides a framework for rational strain engineering strategies aimed at tuning, rather than maximizing, flocculation performance.

## Data Availability

The numerical data underlying all figures and supplemental figures are publicly available in the 4TU Research Data Repository (https://data.4tu.nl/; https://doi.org/10.4121/c023585a-f2a7-4c8d-8a65-eb3da7230020). Transcriptome data of *S. pastorianus* CBS 1483 are available under BioProject accession number PRJNA1441968 at the NCBI BioProject database (https://www.ncbi.nlm.nih.gov/bioproject). Genome sequencing data for strain IMX3185 (*Sp*cI-*flo9-1*Δ::NatNT2 *Sp*cI-*flo9-2*Δ::KanMX) are available under accession number PRJNA1441332.

## SUPPLEMENTAL MATERIAL

Supplemental material aem.00883-26-s0001.docx Fig. S1 to S4; Tables S1, S2, S3, and S5.

Table S4 aem.00883-26-s0002.xlsx Consensus sequence of repeating motifs identified in each Flo protein.

ASM does not own the copyrights to Supplemental Material that may be linked to, or accessed through, an article. The authors have granted ASM a non-exclusive, world-wide license to publish the Supplemental Material files. Please contact the corresponding author directly for reuse.
