# Supplementary material for: Moderate expression and activity of flocculins underlie the characteristic flocculation phenotype of Saccharomyces pastorianus
Source: Appl Environ Microbiol. 2026 Sep 8;92(10):e00883-26. doi: 10.1128/aem.00883-26 (PMC13688867; doi:10.1128/aem.00883-26)
Supplement: Supplementary file 1 [file aem.00883-26-s0001.docx]

# Supplementary information

# Moderate expression and activity of flocculins underlie the characteristic flocculation phenotype of *Saccharomyces pastorianus*

Running title: Flocculin activity and expression shape lager yeast flocculation

Ignacia D. G. L., Bennis N. X., Carqueija Cardoso C., Potma A. A. J., Knibbe E., de Groot P.A., Westerlaken I., van den Broek M., Daran J-M. G.

Department of Biotechnology, Delft University of Biotechnology, van der Maasweg 9, 2629HZ Delft, The Netherlands

Name	email	ORCID

Denzel Ignacia	[D.G.L.Ignacia@tudelft.nl](mailto:D.G.L.Ignacia@tudelft.nl)	0009-0006-8234-529X

Nicole X. Bennis	[N.X.Bennis@tudelft.nl](mailto:N.X.Bennis@tudelft.nl)	0000-0002-3403-0844

Clara Carqueija Cardoso	[D.C.CarqueijaCardoso@tudelft.nl](mailto:D.C.CarqueijaCardoso@tudelft.nl)

Ewout Knibbe	[E.knibbe@tudelft.nl](mailto:E.knibbe@tudelft.nl)	0009-0007-0835-1285

Aike A. J. Potma	[A.A.J.Potma@student.tudelft.nl](mailto:A.A.J.Potma@student.tudelft.nl)

Philip A. de Groot	[P.A.deGroot-1@tudelft.nl](mailto:P.A.deGroot-1@tudelft.nl)

Ilja Westerlaken	[iljawesterlaken@gmail.com](mailto:iljawesterlaken@gmail.com)

Marcel A. van den Broek	[Marcel.vandenBroek@tudelft.nl](mailto:Marcel.vandenBroek@tudelft.nl)	0000-0002-7734-582X

Jean-Marc G. Daran	[J.G.Daran@tudelft.nl](mailto:J.G.Daran@tudelft.nl)	0000-0003-3136-8193

Keywords: Optogenetics, flocculation, gene expression, *Saccharomyces pastorianus,* lager brewing

# Supplementary Figures

**Figure S1.** Alignment of *Spc*I-Flo9-2_1 and *Spc*I-Flo9-2_2 (**A**), and *Spe*I-Flo1-3_1 and *Spe*I-Flo-3_2 (**B**) in Clone Manager 9. The difference in size between *Spc*I-Flo9-2_1 and *Spc*I-Flo9-2_2 was mainly due to *Spc*I-Flo9-2_1 containing 6 repeat type 1 motifs, while *Spc*I-Flo9-2_2 only contained 1. *Spe*I-Flo1-3_1 and *Spe*I-Flo-3_2 were the same length.

**Figure S2.** Flo protein architecture. Relevant domain and motifs were marked. Proteins are grouped according to the sequence alignment in figure 1, with the numbers on the right corresponding to the groups identified.

**Figure S3**. Structural alignment of PA14 domains of the *S. cerevisiae* CEN.PK113-7D and *S. pastorianus* CBS1483 flocculins using US-align (Zhang et al., 2022). All PA14- domain comparisons showed a TM-value greater than 0.80, indicating high structural conservation. The flocculins *Sc*XI-Flo10, *Spe*VI-Flo10 and *Spe*XII-Flo1-5 showed the lowest overall TM-score, indicating distinct structural features aligning with the observed variations in amino acid sequence.

**Figure S4.** Microscopy images of flocculating strains at 400x magnification. The *FLO* -null strain (IMX2897) and CEN.PK113-7D exhibited limited to no aggregation. IMX3012, IMX3101 and IMX3102 showed relatively smaller flocs compared to IMX2912. IMX3013 showed an increased frequency of small cell aggregates compared to CEN.PK113-7D and IMX2897. The white bar represents 50 μm. The data for CEN.PK113-7D, IMX2987 and IMX2912 was derived from (Ignacia et al., 2025).

# Supplementary Tables

**Table S1.** Primers used in this study.

| Primers | Sequence (5’ -> 3’) | Purpose |
| --- | --- | --- |
| 20407 | TCTATAGACACACAAACACAAATACACACACTAAATTAATAGCGGCCGCTAAAATCATGTCTATACCCCATTCC | Amplicon Sequencing of *Spc*VI-*FLO1-1* |
| 20408 | TTTTTTTATAACTTATTTAATAATAAAAATCATAAATCATAAGAAATTCGCTGATCACTAAGACGTGATAGAGCTG | Amplicon Sequencing of *Spc*VI-*FLO1-1* |
| 20918 | TCTATAGACACACAAACACAAATACACACACTAAATTAATAGCGGCCGCTAAAATCATGCCCACGCCTTATTACTGCATA | Repair for integration of *Spe*I-*FLO1-3* into OptoQ-AMP5 |
| 20411 | TCTATAGACACACAAACACAAATACACACACTAAATTAATAGCGGCCGCTAAAATCATGAGAGGTTTGACCCTTATTACG | Repair for integration of *Spe*VI-*FLO12* into OptoQ-AMP5 |
| 20412 | TTTTTTTATAACTTATTTAATAATAAAAATCATAAATCATAAGAAATTCGCTGATCATTAAACAATTGCGAGCAATATGG | Repair for integration of *Spe*VI-*FLO12* into OptoQ-AMP5 |
| 20413 | TCTATAGACACACAAACACAAATACACACACTAAATTAATAGCGGCCGCTAAAATCATGACAATGCCTCATCGC | Repair for integration of *Spc*I-*FLO9-1* into OptoQ-AMP5 |
| 20414 | TTTTTTTATAACTTATTTAATAATAAAAATCATAAATCATAAGAAATTCGCTGATCACTATAAGCGTGATGATAGAA | Repair for integration of *Spc*I-*FLO9-1* into OptoQ-AMP5 |
| 20745 | TCTATAGACACACAAACACAAATACACACACTAAATTAATAGCGGCCGCTAAAATCATGCCTATCCATCATCG | Repair for integration of *Spc*VI-*FLO10* into OptoQ-AMP5 |
| 20746 | TTTTTTTATAACTTATTTAATAATAAAAATCATAAATCATAAGAAATTCGCTGATCATTAAATGATTGCCAATAATACGG | Repair for integration of *Spc*VI-*FLO10* into OptoQ-AMP5 |
| 20747 | TCTATAGACACACAAACACAAATACACACACTAAATTAATAGCGGCCGCTAAAATCATGCCTATCTATAATTATTTATTCTC | Repair for integration of *Spc*XII-*FLO1-5* into OptoQ-AMP5 |
| 20748 | TTTTTTTATAACTTATTTAATAATAAAAATCATAAATCATAAGAAATTCGCTGATCATCAAAAGAATGCCAGCAATATGGAGG | Repair for integration of *Spc*XII-*FLO1-5* into OptoQ-AMP5 |
| 21653 | TACTTCTATAGACACACAAACACAAATACACACACTAAATTAATAGCGGCCGCTAAAATCATGTCTCGTCATTACATATTTTCGACG | Amplicon sequencing of *Spe*XI-*FLO1-4* |
| 21654 | TATTTTTTTTATAACTTATTTAATAATAAAAATCATAAATCATAAGAAATTCGCTGATCATCAAATAATTGCCAGCAGCATGGAG | Amplicon sequencing of *Spe*XI-*FLO1*-4 *RV*/Repair for integration of *Spe*I-*FLO1*-3 into OptoQ-AMP5 |
| 21652 | TATTTTTTTTATAACTTATTTAATAATAAAAATCATAAATCATAAGAAATTCGCTGATCATTACCACACGATTGCCAGCA | Repair for integration of *Spc*I-*FLO9-2* into OptoQ-AMP5 |
| 21679 | TACTTCTATAGACACACAAACACAAATACACACACTAAATTAATAGCGGCCGCTAAAATCATGTCTCTGGCACATTATTGCTTAC | Repair for integration of *Spc*I-*FLO9-2* into OptoQ-AMP5 |
| 14455 | AAAGCAACCTGACCTACAGG | Diagnosis *FLO* insertion in OptoQ-AMP5 |
| 19907 | CCCTGGGTGCAATTTCAGTG | Diagnosis *FLO* insertion in OptoQ-AMP5 |
| 12150 | GCAAACAGTGCCTTCTGTTC | Nested *Spe*I-*FLO1*-3 FW |
| 19618 | ATGCCGTCTCAGGTCTCAGGATTCAAATAATTGCCAGCAGC | Nested (amplicon) *Spe*I-*FLO1*-2/*Spe*I-*FLO1*-3 RV |
| 20961 | TTCGCGTTCAAATTATTTCAC | Nested (amplicon) sequencing *Spe*I-*FLO1*-2 FW |
| 12145 | TTCTTCCGGCCCAAAAAATG | Nested *Spc*I-*FLO9*-2 FW |
| 12146 | GCTGGTTATGCGAAACTGAG | Nested *Spc*I-*FLO9*-2 RV |
| 21638 | GACGGAGAATACGTAGGCTGACTTTTTCGTCAGTTTGTTGTCCGTTTACAAAATTGGTGAGCATAGGCCACTAGTGGATCTG | Repair for integration of KanMX cassette into *Spc*I-*FLO9-1* |
| 21639 | GAAATGTGTAAACACTATAAGCGTGATGATAGAATTATTATATAAGATGATGCCGTTCGTACCTTCGTACGCTGCAGGTC | Repair for integration of KanMX cassette into *Spc*I-*FLO9-1* |
| 21646 | CAGGTACGCTCTTTAAATTTGGAATTTAAAAGGAACAATTGTACATTAAAAGCCCCAACAGCATAGGCCACTAGTGGATCTG | Repair for integration of KanMX cassette into *Spc*I-*FLO9-2* |
| 21647 | AAATGTGTAAACACTATAAGCGTGATGATAGAATTATTAATATAAGATGATGCCGTTCGTACCTTCGTACGCTGCAGGTC | Repair for integration of KanMX cassette into *Spc*I-*FLO9-2* |
| 22083 | GACGGAGAATACGTAGGCTGACTTTTTCGTCAGTTTGTTGTCCGTTTACAAAATTGGTGAGACATGGAGGCCCAGAATACC | Repair for integration of NatNT cassette into *Spc*I-*FLO9-1* |
| 22084 | GAAATGTGTAAACACTATAAGCGTGATGATAGAATTATTATATAAGATGATGCCGTTCGTCCGGTAGAGGTGTGGTCAATAAG | Repair for integration of NatNT cassette into *Spc*I-*FLO9-1* |
| 11200 | ATGTTACATGCGTACACGCG | Diagnosis insertion gRNA in pUDP002 |
| 11201 | ACACGCTTTTTCAGTTCGAG | Diagnosis insertion gRNA in pUDP002 |
| 21615 | CCGACACATGATACTTATCAC | Diagnosis knockout of *Spc*I-*FLO9-1* |
| 21616 | TGCAAAGCCGAATAGTTAG | Diagnosis knockout of *Spc*I-*FLO9-1* |
| 19078 | AGGATATCGTACCACATTTC | Diagnosis knockout of *Spc*I-*FLO9-2* |
| 3811 | CTCGGTGAGTTTTCTCCTTCAT | Diagnosis of insertion KanMX |
| 19903 | TCCGATTCGTCGTCCGATTC | Diagnosis of insertion NatNT2 |

**Table S2.**	Sequencing data accession numbers at NCBI repositories (https://www.ncbi.nlm.nih.gov/). The data are collected under the Bioproject XXX. During the reviewing process the data are available through the following URL:

| Strain | Bioproject | Biosample | | SRA | SRX | Reference |
| --- | --- | --- | --- | --- | --- | --- |
| Transcriptomics |  | |  |  |  |  |
| CBS 1483 | PRJNA1441968 | | SAMN10948597 | [SRR38300359](https://dataview.ncbi.nlm.nih.gov/object/SRR38300359) | SRX33135993 | This study |
|  |  | |  | [SRR38300354](https://dataview.ncbi.nlm.nih.gov/object/SRR38300354) | SRX33135998 |  |
|  |  | |  | [SRR38300353](https://dataview.ncbi.nlm.nih.gov/object/SRR38300353) | SRX33135999 |  |
|  |  | |  | [SRR38300349](https://dataview.ncbi.nlm.nih.gov/object/SRR38300349) | SRX33136003 |  |
|  |  | |  | [SRR38300357](https://dataview.ncbi.nlm.nih.gov/object/SRR38300357) | SRX33135995 |  |
|  |  | |  | [SRR38300350](https://dataview.ncbi.nlm.nih.gov/object/SRR38300350) | SRX33136002 |  |
|  |  | |  | [SRR38300358](https://dataview.ncbi.nlm.nih.gov/object/SRR38300358) | SRX33135994 |  |
|  |  | |  | [SRR38300346](https://dataview.ncbi.nlm.nih.gov/object/SRR38300346) | SRX33136006 |  |
|  |  | |  | [SRR38300351](https://dataview.ncbi.nlm.nih.gov/object/SRR38300351) | SRX33136001 |  |
|  |  | |  | [SRR38300352](https://dataview.ncbi.nlm.nih.gov/object/SRR38300352) | SRX33136000 |  |
|  |  | |  | [SRR38300356](https://dataview.ncbi.nlm.nih.gov/object/SRR38300356) | SRX33135996 |  |
|  |  | |  | [SRR38300348](https://dataview.ncbi.nlm.nih.gov/object/SRR38300348) | SRX33136004 |  |
|  |  | |  | [SRR38300347](https://dataview.ncbi.nlm.nih.gov/object/SRR38300347) | SRX33136005 |  |
|  |  | |  | [SRR38300355](https://dataview.ncbi.nlm.nih.gov/object/SRR38300355) | SRX33135997 |  |
| Genome sequencing |  | |  |  |  |  |
| CBS 1483  [GCA_011022315.1](https://www.ncbi.nlm.nih.gov/datasets/genome/GCA_011022315.1/) | [PRJNA522669](https://www.ncbi.nlm.nih.gov/bioproject/PRJNA522669) | | SAMN10948597 | [SRR8648845](https://trace.ncbi.nlm.nih.gov/Traces?run=SRR8648845)  [SRR8648844](https://trace.ncbi.nlm.nih.gov/Traces?run=SRR8648844)  [SRR8648843](https://trace.ncbi.nlm.nih.gov/Traces?run=SRR8648843)  [SRR8648840](https://trace.ncbi.nlm.nih.gov/Traces?run=SRR8648840)  [SRR8648839](https://trace.ncbi.nlm.nih.gov/Traces?run=SRR8648839) | [SRX5446618](https://www.ncbi.nlm.nih.gov/sra/SRX5446618)  [SRX5446619](https://www.ncbi.nlm.nih.gov/sra/SRX5446619%5baccn%5d)  [SRX5446620](https://www.ncbi.nlm.nih.gov/sra/SRX5446620%5baccn%5d)  [SRX5446623](https://www.ncbi.nlm.nih.gov/sra/SRX5446623%5baccn%5d)  [SRX5446624](https://www.ncbi.nlm.nih.gov/sra/SRX5446624%5baccn%5d) | (Salazar et al., 2019) |
| IMX3185 | PRJNA1441332 | | SAMN56682530 |  |  | This study |

**Table S3**. Percent identity matrix of all flocculins identified in *S. cerevisiae* CEN.PK113-7D, *S. eubayanus* CDFM21L.1 and *S. pastorianus* CBS 1483.

|  | *Sc*I-Flo9 | *Spc*I*-*Flo9-2_1 | *Spc*I*-*Flo9-2_2 | *Spc*I*-*Flo9-1 | *Sc*VIII*-*Flo5 | *Sc*I-Flo1 | *Se*I*-*Flo1-2 | *Spe*I-Flo1-3_2 | *Spe*I-Flo1-3_1 | *Se*I*-*Flo1-1 | *Se*VI*-*Flo10 | *Spe*VI*-*Flo10 | *Se*XII*-*Flo1-4 | *Spe*XII*-*Flo1-5 | *Sc*XI*-*Flo10 | *Se*VI*-*Flo12 | *Spe*VI*-*Flo12 | *Se*IX-Flo11 | *Spe*IX*-*Flo11-2 | *ScI*X-Flo11 |
| --- | --- | --- | --- | --- | --- | --- | --- | --- | --- | --- | --- | --- | --- | --- | --- | --- | --- | --- | --- | --- |
| *Sc*I-Flo9 | 100 | 97,1 | 89,9 | 87,3 | 82 | 85,7 | 61,4 | 61,2 | 61,6 | 60,3 | 40,8 | 45 | 39,9 | 39,3 | 41,6 | 22,4 | 22,9 | 19,7 | 20 | 21,7 |
| *Spc*I*-*Flo9-2_1 | 97,1 | 100 | 91 | 94 | 80,2 | 84,9 | 62,4 | 62,3 | 62,7 | 61,4 | 40,3 | 42,6 | 37,2 | 38,8 | 39,7 | 20,6 | 22,1 | 17,7 | 18,7 | 20,6 |
| *Spc*I*-*Flo9-2_2 | 89,9 | 91 | 100 | 86,2 | 76,4 | 78,3 | 56 | 55,9 | 56,3 | 55 | 42,8 | 47,4 | 40,9 | 41,3 | 43,6 | 22,6 | 22,6 | 20,1 | 19,9 | 21,3 |
| *Spc*I*-*Flo9-1 | 87,3 | 94 | 86,2 | 100 | 82,2 | 88,1 | 62,5 | 62,4 | 62,8 | 62,5 | 40,1 | 44,4 | 38,6 | 39,4 | 41,6 | 20,9 | 22,3 | 18,5 | 19,5 | 22,3 |
| *Sc*VIII*-*Flo5 | 82 | 80,2 | 76,4 | 82,2 | 100 | 89 | 63,7 | 63,6 | 64 | 64,3 | 40,4 | 42 | 37,6 | 38,8 | 40 | 19,5 | 21,7 | 18,3 | 19,3 | 20,4 |
| *Sc*I-Flo1 | 85,7 | 84,9 | 78,3 | 88,1 | 89 | 100 | 64,7 | 64,5 | 64,7 | 65,5 | 45,1 | 46 | 41,8 | 42,9 | 41,9 | 23,6 | 24,3 | 21,4 | 21,6 | 22,8 |
| *Se*I*-*Flo1-2 | 61,4 | 62,4 | 56 | 62,5 | 63,7 | 64,7 | 100 | 99,9 | 97,9 | 95,5 | 51,5 | 58,2 | 55,4 | 55,5 | 47,3 | 21,8 | 23,9 | 17,1 | 18,1 | 18,2 |
| *Spe*I-Flo1-3_2 | 61,2 | 62,3 | 55,9 | 62,4 | 63,6 | 64,5 | 99,9 | 100 | 97,7 | 95,4 | 51,4 | 58 | 55,2 | 55,4 | 47,1 | 21,6 | 23,8 | 17,1 | 18,1 | 18,2 |
| *Spe*I-Flo1-3_1 | 61,6 | 62,7 | 56,3 | 62,8 | 64 | 64,7 | 97,9 | 97,7 | 100 | 93,4 | 52,7 | 60 | 56 | 55,9 | 47,4 | 22,2 | 24,3 | 17 | 17,9 | 18,6 |
| *Se*I*-*Flo1-1 | 60,3 | 61,4 | 55 | 62,5 | 64,3 | 65,5 | 95,5 | 95,4 | 93,4 | 100 | 51,1 | 57,8 | 55 | 55,1 | 47,4 | 21,5 | 23,6 | 17,1 | 18,1 | 18,3 |
| *Se*VI*-*Flo10 | 40,8 | 40,3 | 42,8 | 40,1 | 40,4 | 45,1 | 51,5 | 51,4 | 52,7 | 51,1 | 100 | 99,8 | 46,2 | 46,6 | 35,2 | 18,2 | 19,3 | 15,4 | 16 | 19,3 |
| *Spe*VI*-*Flo10 | 45 | 42,6 | 47,4 | 44,4 | 42 | 46 | 58,2 | 58 | 60 | 57,8 | 99,8 | 100 | 51,3 | 53,2 | 35 | 20 | 20,9 | 17,8 | 18,8 | 20,8 |
| *Se*XII*-*Flo1-4 | 39,9 | 37,2 | 40,9 | 38,6 | 37,6 | 41,8 | 55,4 | 55,2 | 56 | 55 | 46,2 | 51,3 | 100 | 86,9 | 35,8 | 19,6 | 22,5 | 15 | 15,9 | 17,4 |
| *Spe*XII*-*Flo1-5 | 39,3 | 38,8 | 41,3 | 39,4 | 38,8 | 42,9 | 55,5 | 55,4 | 55,9 | 55,1 | 46,6 | 53,2 | 86,9 | 100 | 37,4 | 19 | 21,4 | 16,1 | 16 | 18 |
| *Sc*XI*-*Flo10 | 41,6 | 39,7 | 43,6 | 41,6 | 40 | 41,9 | 47,3 | 47,1 | 47,4 | 47,4 | 35,2 | 35 | 35,8 | 37,4 | 100 | 19,7 | 20,2 | 16,8 | 17,8 | 18,4 |
| *Se*VI*-*Flo12 | 22,4 | 20,6 | 22,6 | 20,9 | 19,5 | 23,6 | 21,8 | 21,6 | 22,2 | 21,5 | 18,2 | 20 | 19,6 | 19 | 19,7 | 100 | 99,5 | 15,2 | 15,6 | 15 |
| *Spe*VI*-*Flo12 | 22,9 | 22,1 | 22,6 | 22,3 | 21,7 | 24,3 | 23,9 | 23,8 | 24,3 | 23,6 | 19,3 | 20,9 | 22,5 | 21,4 | 20,2 | 99,5 | 100 | 15,4 | 16 | 16,1 |
| *Se*IX-Flo11 | 19,7 | 17,7 | 20,1 | 18,5 | 18,3 | 21,4 | 17,1 | 17,1 | 17 | 17,1 | 15,4 | 17,8 | 15 | 16,1 | 16,8 | 15,2 | 15,4 | 100 | 95,3 | 63,3 |
| *Spe*IX*-*Flo11-2 | 20 | 18,7 | 19,9 | 19,5 | 19,3 | 21,6 | 18,1 | 18,1 | 17,9 | 18,1 | 16 | 18,8 | 15,9 | 16 | 17,8 | 15,6 | 16 | 95,3 | 100 | 62,1 |
| *ScI*X-Flo11 | 21,7 | 20,6 | 21,3 | 22,3 | 20,4 | 22,8 | 18,2 | 18,2 | 18,6 | 18,3 | 19,3 | 20,8 | 17,4 | 18 | 18,4 | 15 | 16,1 | 63,3 | 62,1 | 100 |

**Table S4**. Consensus sequence of repeating motifs identified in each Flo protein.

(see file)

**Table S5**. Average rpkm values of full length *FLO* genes in CBS 1483 during a 17 °P wort fermentation in E.B.C. tubes.

| **Chr** | **Strand** | **Length** | **sysname** | **Gene** | **Day 1** | **Day 2** | **Day 5** | **Day 6** | **Day 9** | **Day 12** | **Day 15** |
| --- | --- | --- | --- | --- | --- | --- | --- | --- | --- | --- | --- |
| *Spc*I | + | 3012 | YHR211W | *Spc*I-*FLO9*-1 | 2,6 | 2,3 | 10,0 | 10,2 | 7,4 | 5,1 | 10,3 |
| *Spc*I | - | 3132 | YHR211W | *Spc*I-*FLO9*-2_1 | 4,0 | 4,2 | 19,0 | 18,7 | 12,4 | 10,3 | 19,3 |
| *Spc*I | - | 2610 | YAL063C | *Spc*I-*FLO9*-2_2 | 0,7 | 0,9 | 0,6 | 1,1 | 2,0 | 1,1 | 1,6 |
| *Spe*I | + | 2367 | YHR211W | *Spe*I-*FLO1*-2 | 3,9 | 3,8 | 8,6 | 17,3 | 6,6 | 5,6 | 8,1 |
| *Spe*I | - | 2100 | YAL063C | *Spe*I-*FLO1*-3 | 5,0 | 4,9 | 8,9 | 16,0 | 7,2 | 4,8 | 9,2 |
| *Spe*VI | - | 3378 | YKR102W | *Spe*VI-*FLO10* | 38,5 | 4,7 | 3,7 | 9,1 | 5,1 | 4,0 | 12,8 |
| *Spe*VI | - | 3765 | YHR211W | *Spe*VI-*FLO12* | 1,5 | 0,5 | 0,2 | 0,7 | 0,6 | 0,6 | 1,9 |
| *Spe*XII | - | 2892 | YAL063C | *Spe*XII-*FLO1*-5 | 0,5 | 0,7 | 1,4 | 2,3 | 2,5 | 1,6 | 2,6 |
| *Spc*II | - | 1671 | YBR298C | *Spc*II-*MAL31* | 337,2 | 374,7 | 328,5 | 743,9 | 205,7 | 353,3 | 810,3 |
| *Spc*IV | - | 747 | YDR050C | *Spc*IV-*TPI1* | 8013,3 | 6393,0 | 1772,7 | 599,8 | 542,7 | 462,7 | 987,4 |
| *Spe*III-*c*III | + | 1251 | YCR012W | *Spe*III-*c*III-*PGK1* | 5828,0 | 5468,0 | 2905,1 | 1106,8 | 615,8 | 388,2 | 848,6 |
